# Association of copy number variation across the genome with neuropsychiatric traits in the general population

**DOI:** 10.1002/ajmg.b.32637

**Published:** 2018-04-24

**Authors:** Anna L. Guyatt, Evie Stergiakouli, Joanna Martin, James Walters, Michael O'Donovan, Michael Owen, Anita Thapar, George Kirov, Santiago Rodriguez, Dheeraj Rai, Stan Zammit, Tom R. Gaunt

**Affiliations:** ^1^ MRC Integrative Epidemiology Unit, Population Health Sciences, University of Bristol Bristol United Kingdom; ^2^ Centre for Academic Mental Health, Population Health Sciences, University of Bristol Bristol United Kingdom; ^3^ MRC Centre for Neuropsychiatric Genetics and Genomics Cardiff University Cardiff United Kingdom; ^4^ Department of Medical Epidemiology & Biostatistics Karolinska Institutet Stockholm Sweden

**Keywords:** ALSPAC, childhood, genetic epidemiology, structural variation

## Abstract

Copy number variants (CNVs) are associated with psychiatric conditions in clinical populations. The relationship between rare CNV burden and neuropsychiatric traits in young, general populations is underexplored. A total of 6,807 children from the Avon Longitudinal Study of Parents and Children (ALSPAC) were studied. CNVs were inferred from single nucleotide polymorphism‐array data using PennCNV. After excluding children with known candidate CNVs for schizophrenia (SCZ), rare (<1%) CNV burden (total number of genes affected by CNVs, total length of CNVs, and largest CNV carried) was analyzed in relation to: psychotic experiences (PEs) and anxiety/depression in adolescence; autism spectrum disorder (ASD) and attention‐deficit hyperactivity disorder (ADHD), ASD and ADHD traits, and cognitive measures during childhood. Outcomes were also assessed in relation to known SCZ CNVs. The number of genes affected by rare CNVs was associated with a continuous measure of ASD: the standardized mean difference [SMD] per gene affected was increased by 0.018 [95%CI 0.011,0.025], *p* = 3e‐07 for duplications and by 0.021 [95%CI 0.010, 0.032], *p* = 1e‐04 for deletions. In line with our published results on educational attainment in ALSPAC, intelligence quotient (IQ) was associated with CNV burden: the SMD per gene affected was −0.017 [95%CI −0.025, −0.008] *p* = 1e‐04 for duplications and −0.023 [95%CI −0.037, −0.009], *p* = .002 for deletions. Associations were also observed for measures of coherence, attention, memory, and social cognition. SCZ‐associated deletions were associated with IQ (SMD: −0.617 [95%CI −0.936, −0.298], *p* = 2e‐04), but not with PEs or other traits. We found that rare CNV burden and known SCZ candidate CNVs are associated with neuropsychiatric phenotypes in a nonclinically ascertained sample of young people.

## 1 INTRODUCTION

The heritability of neuropsychiatric traits is substantial (Polderman et al., 2015): twin studies estimate the heritability of schizophrenia (SCZ) to be 0.81 (95%CI 0.73, 0.90) (Sullivan Sullivan, Kendler, & Neale, 2003), with estimates of a similar magnitude (0.75–0.80) for autism spectrum disorder (ASD) (Ronald & Hoekstra, 2011), intelligence quotient (IQ) (Plomin & Deary, 2015), bipolar disorder (BPD) (Sullivan, Daly, & O'Donovan, 2012), and attention‐deficit hyperactivity disorder (ADHD) (Faraone et al., 2015). Other disorders, such as major depressive disorder (MDD) may be less heritable (0.37, [95%CI 0.31, 0.42]) (Sullivan, Neale, & Kendler, 2000), although there is variance in estimates by subtype (e.g., recurrence, age of onset) (Ripke et al., 2013). While the magnitude of heritability estimates varies by the methodology used (e.g., population‐based designs estimate lower heritability of SCZ (0.67 [95%CI 0.64, 0.71]) (Samson & Wong, 2015; Wray & Gottesman, 2012) and ASD (0.50 [95%CI 0.45–0.56]) (Sandin et al., 2014), respectively, in population‐based designs) (Sullivan et al., 2003), genetic factors are undoubtedly important contributors to variance observed in neuropsychiatric traits.

It is known that rare copy number variants (CNVs) contribute to the etiology of many neuropsychiatric phenotypes, including (but not limited to) SCZ (Grozeva et al., 2010; Kirov, 2015; Kirov et al., 2009, 2012; Morrow, 2010; Stone et al., 2008), ASDs (Ching et al., 2010; Glessner et al., 2009; Morrow, 2010; Sebat et al., 2007; Urraca et al., 2013), ADHD (Martin et al., 2014; Williams et al., 2010), cognitive ability (Guffanti et al., 2013), and educational attainment (Männik et al., 2015). A useful working definition of CNVs is that they are genomic regions (often classified as >1 kb in length) that are duplicated or deleted in comparison to a reference genome (Redon et al., 2006). Compared to phenotype associations with single nucleotide variants, CNVs tend to confer larger effects (Girirajan et al., 2011; Thapar & Cooper, 2013). A recent survey of rare CNVs in 60,000 human exomes found that ∼70% of individuals carry at least one rare, genic CNV, and that on average, copy number gains are more common than losses (Ruderfer et al., 2016).

There is substantial pleiotropy between many of these psychiatric phenotypes: in a study of five disorders (SCZ, BPD, MDD, ASD, ADHD), consistent genetic correlations (*rG*) estimated from single nucleotide polymorphism (SNP) data by both restricted maximum likelihood) and linkage disequilibrium‐score regression were found, including the highest *rG* of ∼0.79 for SCZ and BPD (Bulik‐Sullivan et al., 2015). This same study observed more modest (yet still substantial) *rG* values of 0.14 and 0.23 for SCZ/ASD and SCZ/ADHD. Cross‐phenotypic overlap is also observed with CNV associations: perhaps the strongest evidence comes from CNVs robustly associated with SCZ, which are also associated with developmental delay, ASD, and congenital malformations (Kirov et al., 2014; O'Donovan & Owen, 2016). CNVs implicated in ASD and SCZ have also shown enrichment in children with ADHD (Williams et al., 2010).

While many studies have examined the association of genetic variation in relation to clinical psychiatric diagnoses, genetic association studies of neuropsychiatric traits in population‐based samples of younger individuals (i.e., prior to the age of onset of many psychiatric conditions) are less numerous. Within the Avon Longitudinal Study of Parents and Children (ALSPAC) cohort, the use of common genetic variation has provided insight into the shared etiology of psychiatric disorders and traits in the general population. Salient examples include: the genetic correlation between ADHD traits and ADHD diagnosis (Stergiakouli et al., 2015), the shared genetic risk of ASD and population‐level variance in social and communication ability (Robinson et al., 2016), and the association of a polygenic risk score for SCZ with adolescent measures of negative symptoms and anxiety (but not psychotic experiences [PEs]) (Jones et al., 2016) SCZ also shows a strong genetic correlation with ADHD in ALSPAC (Nivard et al., 2017). Concerning rarer variation, in a large study of the burden of rare CNVs and cognitive phenotypes in unselected populations, including ALSPAC, we previously found that these variants were negatively associated with educational attainment (Männik et al., 2015). In UK Biobank, carriers of known pathogenic CNVs have also been observed to have reduced cognitive performance (Kendall et al., 2016). However, associations between rare CNVs and other neuropsychiatric traits are underexplored.

Using data from a young, general population sample (ALSPAC), we first sought to study the burden of large, rare CNVs on a range of neuropsychiatric phenotypes, including ADHD, ASD, depression, anxiety, PEs, and neurocognition. The secondary objective was to test the relationship of known SCZ candidate CNVs with the traits studied.

## 2 MATERIALS AND METHODS

### 2.1 Cohort details

The ALSPAC is a prospective cohort of mothers and children. Between 1991 and 1992, 14,541 women living in the former county of Avon, UK were recruited during pregnancy, of whom 13,761 were enrolled into the study. Participants have been followed up longitudinally since recruitment. Further details are available in the cohort profile papers (Boyd et al., 2013; Fraser et al., 2013), and the study website contains details of available data through a fully searchable data dictionary: http://www.bris.ac.uk/alspac/researchers/data-access/data-dictionary/

Ethical approval for the study was obtained from the ALSPAC Ethics and Law Committee and the Local Research Ethics Committees.

### 2.2 Genotyping and quality control

The ALSPAC children were genotyped on the Illumina HumanHap550‐Quad platform, by the Wellcome Trust Sanger Institute, Cambridge, UK, and the Laboratory Corporation of America, Burlington, NC, using support from 23andMe. Samples were removed if there were gender mismatches, disproportionate heterozygosity, >3% missingness, and insufficient sample replication (IBD <0.8). A total of 8,365 participants who were unrelated at an IBD of >0.125 were included, and all were of European ancestry (non‐Europeans were removed after multidimensional scaling, and comparison with the HapMap CEU population). Genomic locations described in this paper relate to NCBI build 37/hg19, unless otherwise stated.

### 2.3 CNV calling

CNVs were called on 7,572 participants using PennCNV (Wang et al., 2007), and the default libraries provided with the package for the HumanHap550 array (Hidden Markov Model [hh550.hmm] and Population Frequency of B Allele [hh550.hg18.pfb] files). Log R ratio (LRR) and B Allele Frequency were derived from Illumina Final Report files using a custom Python script (available at: https://github.com/MRCIEU/IlluminaFinalRep_LRRBAF).

**Figure 1:**
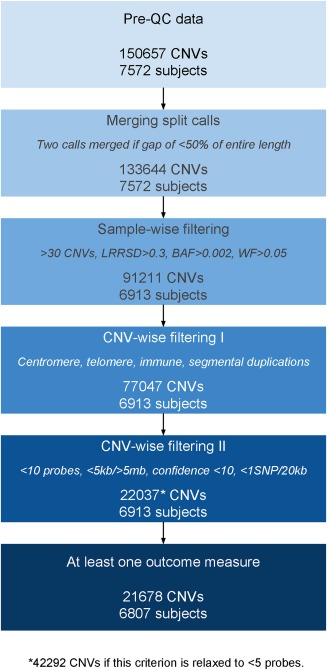
Schematic of the QC process. A schematic of the QC process overall, including the number of individuals and CNVs retained after each step of filtering. Before QC, there were 150,657 CNVs and 7,572 subjects, and after QC, there were 22,037 CNVs and 6,913 subjects (11,965 low‐frequency [<1%] CNVs). After filtering for those with no outcome data, 21,678 CNVs remained (11,750 low‐frequency [<1%] CNVs), split among 6,807 subjects [Color figure can be viewed at http://wileyonlinelibrary.com]

After calling, all 7,572 subjects had at least one CNV (with 150,657 CNVs among this group in total). These individuals were retained for quality control (QC). Figure 1 shows a flowchart of the QC and filtering process for the CNV data. First, CNVs were merged if they were separated by a gap less than half of their combined length using the “clean_cnv.pl” script provided within PennCNV (Wang et al., 2007). Individuals were then dropped from the analysis if they had >30 CNV calls (Nag et al., 2013), if they had a LRR standard deviation (*SD*) of >0.3, a B Allele Frequency drift (BAF drift) of >0.002, or an absolute waviness factor of >0.05. The filter values for LRR *SD*, BAF drift and waviness factor are all as recommended by PennCNV developers (Wang et al., 2007). Next, CNVs were removed from the analysis if at least 50% of the CNV call overlapped with telomeric, centromeric, or immunoglobulin regions (see Supporting Information Notes 1, 2 and 3 for details of coordinates). Known regions of segmental duplications were also removed (as downloaded from the UCSC Genome Browser [https://genome.ucsc.edu/] [Kent et al., 2002]). CNVs were removed if they fulfilled any of the following criteria: if they spanned <10 probes, were <5 kb or >5 Mb in length, or had a confidence score of <10. Finally, those CNVs that had a density of less than one probe per 20 kb were removed.

After QC, there were 22,037 CNVs and 6,913 subjects (311 of whom had no CNVs after QC). PLINK was used to identify CNVs with a sample frequency of <1% (using PLINK flags –cnv‐freq‐exclude‐above 69 [as 6,913 individuals passed QC], –cnv‐overlap 0.5) (Purcell et al., 2007; Tansey et al., 2016).

After filtering for those with no outcome data, but complete confounder data, 21,678 CNVs remained (11,750 with a frequency <1%), split among 6,807 subjects. CNVs were mapped to genes using the “refGene” database, also downloaded from UCSC Genome Browser (Kent et al., 2002).

### 2.4 Phenotypes

**Figure 2:**
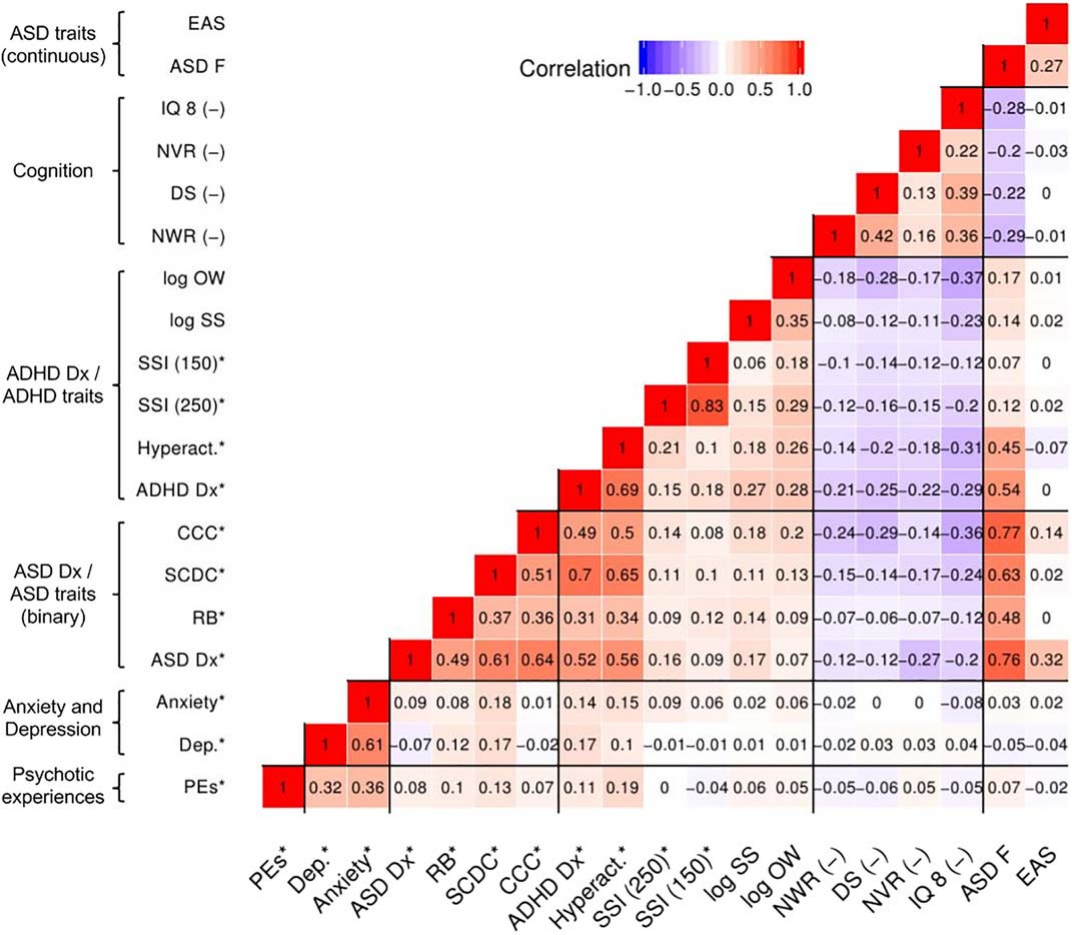
Heatmap of phenotypic correlations between neuropsychiatric traits. This heatmap shows the correlations between all outcomes studied, computed using the “mixed.cor” function provided in the R package “psych.” Pearson correlations are computed for pairs of continuous variables, tetrachoric correlations for dichotomous variables (denoted with a “*”), and biserial correlations for mixed pairs. Positive correlations are given in red, and negative correlations in blue, with intensity indicating the magnitude of the correlation. (–) = lower score is indicative of reduced performance on these metrics (for all other traits, higher scores indicate a reduced performance, or the trait has been dichotomized so that the risk group is coded as “1,” control group as “0”). For expansion of abbreviations, see Table 1

Descriptive statistics of each variable, as well as the numbers in the risk group (or means/medians for continuous variables) are summarized in Table 1. Correlations between phenotypes are shown in Figure 2. See Supporting Information Methods for extended details of the phenotypes. Diagnoses were studied as binary variables. Traits were studied continuously where possible, and dichotomized (defining a risk group as close to 10% as possible) if skewness prohibited simple transformation.

**Table 1:** Summary of neuropsychiatric outcomes studied in this paper

Domain	Binary or continuous?	Trait (abbreviation)	Trait description/age at measurement	Measure	Value	Total *N* (max = 6,807)
Psychosis	Binary	Psychosis‐like symptoms (PEs)	Psychosis‐like experiences recorded (12 or 18 years)	*N* (% risk)	765 (16.9)	4,540
Depression and anxiety (Dep. & Anx.)	Binary	Depression (Dep.)	Symptoms meeting ICD‐10 criteria (18 years)	*N* (% risk)	244 (8.62)	2,830
	Binary	Anxiety (Anxiety)		*N* (% risk)	292 (10.3)	2,830
Autism spectrum disorders (ASDs)	Binary	ASD diagnosis (ASD Dx)	ASD diagnosis in ALSPAC	*N* (% risk)	82 (1.2)	6,807
	Continuous	ASD traits (ASD F)	Mean of seven ASD factors (Steer et al., 2010) (lower = favourable)	Mean (*SD*)	−0.08 (0.33)	6,501
	Continuous	Sociability (EAS)	REFLECTED (1‐variable) Emotionality, Activity and Sociability temperament scale (lower = favourable) (38 months)	Mean (*SD*)	−17.2 (3.1)	5,283
	Binary	Repetitive behaviour (RB)	Repetitive behaviour measure (dichotomized) at 69 months	*N* (% risk)	326 (6.89)	4,730
	Binary	Social communication (SCDC)	Skuse social cognition score (dichotomized) at 91 months	*N* (% risk)	436 (9.35)	4,664
	Binary	Coherence (CCC)	Child coherence scale (dichotomized) at 9 years (108 months)	*N* (% risk)	452 (9.53)	4,742
ADHD (Hyperactivity, inhibition/impulse control, attention)	Binary	ADHD diagnosis (ADHD Dx)	(pseudo‐) ADHD diagnosis in ALSPAC	*N* (% risk)	90 (1.91)	4,714
	Binary	Hyperactivity score (Hyperact.)	Symptom score for hyperactivity (dichotomized) (81 months)	*N* (% risk)	487 (10.3)	4,715
	Binary	Inhibition and impulse control (SSI [250])	Performance on Stop‐Signal Inhibition task (250 millisecond [ms] delay) (dichotomized) (10 years)	*N* (% risk)	430 (9.95)	4,323
	Binary	Inhibition and impulse control (SSI [150])	Performance on Stop‐Signal Inhibition task (150 ms delay) (dichotomized) (10 years)	*N* (% risk)	375 (8.67)	4,323
	Continuous	Selective attention (log SS)	Time (secs): “Sky Search” task (lower = favourable) (8 years)	Median (IQR)	4.82 (4.1–5.76)	4,451
	Continuous	Attention control (log OW)	Time (secs): “Opposite Worlds” task (lower = favourable) (8 years)	Median (IQR)	16.5 (14.5–19)	4,468
Cognition	Continuous	Phonological memory (NWR) (–)	Score on non‐word repetition task (higher = favourable) (8 years)	Mean (*SD*)	7.28 (2.51)	4,573
	Continuous	Working memory (DS) (–)	Score on digit span task (higher = favourable) (8 years)	Mean (*SD*)	13 (2.95)	4,452
	Continuous	Social cognition (NVR) (–)	REFLECTED (1‐variable) Number of errors made on DANVA face recognition (higher = favourable) (8 years)	Mean (*SD*)	−3.56 (2.77)	4,190
	Continuous	IQ 8 (–)	IQ score on WISC III (higher = favourable) (8 years)	Mean (*SD*)	105 (16.3)	4,544

(–) = lower score is indicative of reduced performance on these metrics (for all other traits, higher scores indicate a reduced performance, or the trait has been dichotomized so that the risk group is coded as “1,” control group as “0”). Gray = binary. White = continuous. Abbreviations in this Table are used in subsequent Tables/Figures.

#### 2.4.1 Psychiatric traits and diagnoses

##### Psychotic experiences

The psychosis‐like symptoms (PLIKS) semi‐structured interview was carried out at both 12 and 18 years. The content and reliability of the interview has been described previously (Zammit et al., 2013), but briefly, it covers the occurrence of hallucinations, delusions, and experiences of thought interference, with ratings based on the Schedule for Clinical Assessment in Neuropsychiatry. For this study, the primary outcome was a binary definition of suspected or definite PEs at either age 12 or 18, versus no PEs at these ages.

##### Anxiety and depression

Binary variables for anxiety and depression were derived using scores from the Computerized Interview Schedule—Revised (CIS‐R), carried out at 18 years (Lewis, Pelosi, Araya, & Dunn, 1992). This interview establishes the type and severity of neurotic symptoms, and categorizes depressive episodes according to ICD‐10 criteria (including mild, moderate, or severe depression). Anxiety was defined as the presence of ICD‐10 concordant diagnoses of generalized anxiety disorder, social phobia, specific phobia, agoraphobia, or panic disorder, using the CIS‐R (Jones et al., 2016).

##### ASDs and traits

###### ASD diagnosis

Diagnoses of ASD have been recorded in ALSPAC via several methods, as described previously (Golding et al., 2017): all children given a statement of special educational needs in the Avon area were reviewed to identify those diagnosed as having ASD according to ICD‐10 criteria (Williams, Thomas, Sidebotham, & Emond, 2008). Maternal reports were also used to source cases, according to responses to the question (asked when children were 9 years): “Have you ever been told that your child has autism, Asperger's syndrome or autistic spectrum disorder?.” Additional sources of cases included: children diagnosed by age 16, due to classification by the educational system as requiring special educational needs due to ASD; text responses to ALSPAC questionnaires relating to ASD diagnosis between 6 months and 11 years; and finally, letters from parents to the ALSPAC study director.

##### ASD traits

###### Mean of seven ASD factors generated previously in ALSPAC

Steer, Golding, and Bolton (2010) report a factor analysis of 93 individual measures related to ASD in ALSPAC. They generated seven factors. In the current paper, the mean of these seven factors was used as a global measure of ASD on a continuous scale. While initially very skewed, this variable was readily transformable to approximate normality after reflection and log‐transformation (i.e., after transformation, a higher score is associated with ASD). Histograms of this variable before and after transformation are shown in Supporting Information Figure 1.

###### Specific measures of ASD that predict ASD diagnosis in ALSPAC

Four ASD traits that have previously been noted to form a predictive model of ASD in ALSPAC (pseudo‐*r* ^2^ reported as .48) (Steer et al., 2010) were also used in this analysis. The coherence subscale of the Children's Communication Checklist (CCC), was scored at 9 years (Bishop, 1998), and includes questions such as whether the child could explain the rules of a simple game to a younger child. The Social and Communication Disorders Checklist (SCDC, 91 months) (Sebat et al., 2007) includes questions such as whether the child was able to realize if they had offended people, and about whether they responded to instructions. Another measure explained the presence of repetitive behaviors (RB) at 69 months) (Rutter, Tizard, & Whitmore, 1971). Finally, the sociability subscale of the Emotionality Activity and Sociability (EAS) temperament scale included measures of whether the child enjoyed the company of people at age 38 months (Buss & Plomin, 1984).

The RB and SCDC, CCC measures were highly skewed and were therefore dichotomized, defining 10% of the sample for each group as the “risk” group for ASD traits. The SCDC has previously been dichotomized using a cutoff of 9 (Barona, Kothari, Skuse, & Micali, 2015), and in this paper, the dichotomization method (using a ∼10% risk group) supported a similar cutoff of 8. All traits were based on responses to questionnaires filled out by mothers. The EAS was approximately normally distributed, and thus was analyzed continuously, after reflecting the variable, so that a higher score was associated with increased risk of ASD. Histograms of these variables before and after transformation are shown in Supporting Information Figure 2.

##### ADHD and associated traits

###### ADHD diagnosis

ADHD diagnoses are recorded in ALSPAC according to DSM‐IV criteria, and based on the Development and Well‐Being Assessment at 91 months. This diagnosis variable was a composite of children with combined, inattentive, or hyperactive‐impulsive ADHD diagnoses. Children with pervasive developmental disorders were excluded.

###### Hyperactivity

Hyperactivity was assessed using the hyperactivity score from the Strengths and Difficulties Questionnaire (Goodman, 1997). This measure was derived from the results of a maternal questionnaire administered when children were 81 months old, and provides a quantitative measure of ADHD that can also be used to generate a categorical definition (Stergiakouli, Thapar, Davey Smith, & Pediatr, 2016). Since the measure was zero‐inflated in the participants included in this study (see Supporting Information Figure 3), this variable was analyzed as a dichotomous variable, defining a ∼10% risk group (equivalent to using a cutoff of 7, as previously used in ALSPAC) (Stergiakouli et al., 2016).

**Figure 3:**
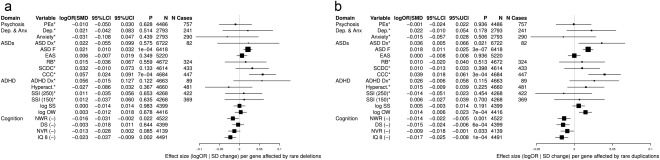
Number of genes affected by rare CNVs in relation to neuropsychiatric traits. This analysis is a regression (logistic for binary traits, linear for continuous traits) of the number of genes affected by rare CNVs (exposure) in relation to neuropsychiatric traits (outcome). This is done separately for deletions (a) and duplications (b). For expansion of trait abbreviations, see Table 1. Other abbreviations: logOR|SMD = effect size (logOR for binary traits [denoted with a *], *SD* change for continuous traits); LCI/UCI = lower and upper bounds of 95% confidence interval; (–) = lower score is indicative of reduced performance on these metrics (for all other traits, higher scores indicate a reduced performance, or the trait has been dichotomized so that the risk group is coded as “1,” control group as “0”). All analyses were adjusted for sex and population ancestry, using the first two principal components generated from the SNP genotype data used to call CNVs

###### Inhibition and impulse control

Inhibition and impulsivity were measured using the “Stop‐Signal Inhibition” task (see Supporting Information Methods) (Logan, 1994; Logan, Cowan, & Davis, 1984; Pindus et al., 2015). The number of trials correct at the 250 ms delay and the 150 ms delay were used in this analysis. Both variables were dichotomized into 10% risk groups, since they were negatively skewed, as shown in Supporting Information Figure 4.

###### Attention

Two measures of attention, capturing selective attention and attentional control were used (both measured at 8 years), and were assessed using the “Sky Search” and “Opposite Worlds” subtasks of the Test of Everyday Attention for Children “TEACh” task (see Supporting Information Methods) (Manly & Thames Valley Test Company, 1999). Both measures were log‐transformed as this approximated a better normal distribution (three observations on the selective attention measure were dropped to facilitate this). For both attentional measures, a higher score indicates reduced attention, since the measures were based on response times. Histograms of these variables before and after transformation are shown in Supporting Information Figure 5.

#### 2.4.2 Neurocognitive traits

See Supporting Information Figure 6 for histograms of cognitive variables. All of these variables were analyzed continuously.

##### Memory

Working memory was assessed using the digit span subtest of the Wechsler Intelligence Scale for Children (WISC), administered at 8 years (Wechsler, Golombok, & Rust, 1992). Phonological memory was assessed using a nonword repetition task that was an adaptation of a previously published task (Gathercole, Willis, Baddeley, & Emslie, 1994). For both of these measures, a higher score indicates better performance. See Supporting Information Methods for details.

##### Social cognition

Social cognition (nonverbal recognition) was measured using the number of errors made on the Diagnostic Analysis of Non‐verbal Accuracy (DANVA) face recognition task (Nowicki & Duke, 1994). This variable was reflected before analysis, so that a higher score indicates a better performance. See Supporting Information Methods for task details.

##### Intelligence quotient

IQ was measured at 8 years by the WISC (Wechsler et al., 1992). A higher score indicates a higher IQ.

### 2.5 Statistical analysis

All analyses were carried out using R. Analyses wherein the number of subjects in a given cell was <5 were censored to protect confidentiality, concordant with ALSPAC policy (https://www.bristol.ac.uk/media-library/sites/alspac/documents/alspac-publications-checklist.pdf).

#### 2.5.1 Transformations of phenotypes and covariates

Psychiatric diagnoses of PEs, anxiety, depression, ASD, and ADHD were binary variables. Due to severe non‐normality, many of the measures were dichotomized as detailed above. To summarize, the transformed mean ASD factor score, the measures of cognition, and attention were analyzed as continuous variables. Continuous outcomes were standardized before analyses, to give mean = 0 and *SD* = 1. All analyses were adjusted for sex and population ancestry, using the first two principal components generated from the SNP genotype data used to call CNVs.

#### 2.5.2 Genome‐wide CNV burden

For all analyses, deletions and duplications were analyzed separately. Effect sizes are presented as *SD* changes (continuous outcomes) and log odds ratios (binary outcomes). In burden analyses, individuals carrying one of the known pathogenic CNVs (described below) were dropped (*n* = 85). Analyses retaining these subjects are available in the Supporting Information.

Analyses were undertaken to assess CNV burden in several ways: in the first analysis, the total number of genes affected by rare CNVs (frequency <1%) was computed for each subject. This variable was used as a measure of rare CNV burden, and each outcome was regressed in turn against it, using either logistic or linear models for binary and continuous outcomes, respectively. We also ran sensitivity analyses, in which the main analysis was repeated, but with individuals carrying known pathogenic CNVs retained.

In addition, we performed two other types of burden analyses, relating to the length of CNVs carried. First, we computed the total length (in kilobases, kb) of deletions and duplications of a frequency <1% per subject. Next, based on the methods of Szatkiewicz et al. (2014) and Männik et al. (2015), the length of the largest rare deletion and duplication carried was noted for each individual. These variables were then categorized into size: the reference group consisted of individuals carrying no rare (<1%) CNVs of >100 kb (or common CNVs only). Three other categories were defined, in which the largest rare CNV carried was >100 kb to ≤500 kb, >500 kb to ≤1 Mb, or >1 Mb. These categories span the breadth of CNV sizes studied in two recent papers of CNV burden in large cohorts (Männik et al., 2015; Szatkiewicz et al., 2014). For each outcome, presence of a CNV in each size category was compared to the reference category.

#### 2.5.3 Candidate CNVs analysis

A number of CNVs have been identified as being associated with SCZ (Kendall et al., 2016). The association between presence of at least one of these 12 rare CNVs (7 deletions, 5 duplications), and each outcome, was assessed separately for deletions, duplications, and any CNV (see Table 2). For the analysis assessing the effect of pathogenic deletions only, those individuals carrying pathogenic duplications were dropped, and vice versa. For coordinates of candidate CNVs, critical regions (defined as stated in Kendall et al. [2016]), and criteria for defining CNVs, see Table 3. To establish the presence or absence of these CNVs, the package “bedtools” was used to compute overlaps between observed CNVs and critical regions (Quinlan & Hall, 2010). For neurexin‐1 (*NRXN1*) deletions, exon coordinates were downloaded from “TableBrowser” and according to transcript NM_004801 (Kent et al., 2002). Results are summarized in Table 4.

**Table 2:** Descriptive statistics of CNVs identified in ALSPAC, in relation to size, and overlap with genes, separately by deletions and duplications

Number of individuals^a^ carrying CNVs of given sizes	*N* (deletions)	*N* (duplications)	*N* (deletion|duplication)
Rare CNVs (excluding individuals with at least one of 12 known pathogenic CNVs^b^)			
Largest rare CNV ≤100 kb, or no rare CNVs (N)	4,942	4,817	3,526
[Largest rare CNV >100 kb (N)]	1,780	1,905	3,196
Largest rare CNV >100 kb to ≤500 kb (N)	1,646	1,521	2,688
Largest rare CNV >500 kb to ≤1 Mb (N)	88	271	351
Largest rare CNV >1 Mb (N)	46	113	157
Total number of subjects	6,722	6,722	6,722
Median length of rare CNVs in kb (IQR)	36 (0–131)	11 (0–136.1)	123 (29–304)
Median number of genes affected by rare CNVs	0 (0–1)	0 (0–1)	1 (0–3)

IQR = interquartile range; kb = kilobase; Mb = megabase; rare = frequency <1%; SCZ = schizophrenia.

^a^Total *N* for whole paper = 6,807, *N* = 6,722 after excluding 85 carriers of pathogenic CNVs (61 deletion carriers, 24 duplication carriers).

^b^See Table 3 for coordinates of these CNVs.

**Table 3:** Coordinates (hg19) of critical regions of 12 SCZ‐associated CNVs

Chr*	Start	End	Location	Type	Likely candidate gene(s)	Criteria for defining this CNV^b^
chr1	146,527,987	147,394,444	1q21.1	Deletion	.	Size >50% of critical region
chr1	146,527,987	147,394,444	1q21.1	Duplication	.	Size >50% of critical region
chr2	50,145,643	51,259,674	2p16.3	Deletion	*NRXN1*	Deletion of at least one exon (NM_004801)
chr3	195,720,167	197,354,826	3q29	Deletion	*DLG1*	Size >50% of critical region
chr7^c^	72,744,915	74,142,892	7q11.23	Duplication	*STX1A*	Size >50% of critical region
chr15	22,805,313	23,094,530	15q11.2	Deletion	*CYFIP1*	Size >50% of critical region
chr15**^c^	23,561,268	28,390,339	15q11.2‐q13.1	Duplication	*UBE3A* and *GABA* receptor gene cluster	Full critical region
chr15	31,080,645	32,462,776	15q13.3	Deletion	.	Size >50% of critical region
chr16	15,511,655	16,293,689	16p13.11	Duplication	*MYH11*	Size >50% of critical region
chr16	21,950,135	22,431,889	16p12.1	Deletion	*UQCRC2*	Size >50% of critical region
chr16	29,650,840	30,200,773	16p11.2	Duplication	*ALDOA, CORO1A, MAPK3, TAOK2*	Size >50% of critical region
chr22^c^	19,037,332	21,466,726	22q11.2	Deletion	*PI4KA, SEPT5*	Size >50% of critical region

Chr = chromosome.

^a^Coordinates taken from Supporting Information of Kendall et al., 2016 (except for **). For analyses, the liftOver tool was used to map from hg19 to hg18 coordinates, specifying minimum ratio of bases that must remap as 0.95.

^b^See Supporting Information Table S4 of Kendall et al. (2016).

^c^CNVs not observed in this study population.

**Table 4:** Analysis of carriage of SCZ candidate genes in relation to neuropsychiatric traits

			Deletion or duplication						Deletion						Duplication					
Domain	Type	Trait	logOR/ SMD	95%LCI	95%UCI	*p*	N	N1^b^	logOR/SMD	95% LCI	95% UCI	*p*	N	N1^b^	logOR/ SMD	95% LCI	95% UCI	*p*	N	N1^b^
Psychosis	Binary	PLIKS any^a^	−0.153	−0.909	0.603	.691	4,540	8												
Dep. & Anx.	Binary	Dep.^a^																		
	Binary	Anxiety^a^																		
ASDs	Binary	ASD Dx^a^																		
	Cont.	ASD F	0.311	0.102	0.520	.003	6,501	83	0.369	0.124	0.614	.003	6,478	60	0.161	−0.234	0.556	.425	6,441	23
	Cont.	EAS^a^	0.153	−0.094	0.401	.225	5,283	63	0.342	0.052	0.631	.021	5,266	46	−0.357	−0.832	0.118	.141	5,237	17
	Binary	RB^a^																		
	Binary	SCDC^a^																		
	Binary	CCC^a^	−0.074	−1.001	0.853	.876	4,742	5												
ADHD	Binary	ADHD Dx^a^																		
	Binary	Hyperact.^a^	0.084	−0.777	0.946	.848	4,715	6												
	Binary	SSI (250)^a^	0.442	−0.315	1.199	.252	4,323	8												
	Binary	SSI (150)^a^	0.267	−0.589	1.122	.542	4,323	6												
	Cont.	log SS	0.227	−0.04	0.494	.096	4,451	52	0.296	−0.02	0.612	.066	4,436	37	0.054	−0.442	0.549	.832	4,414	15
	Cont.	log OW	0.230	−0.043	0.502	.099	4,468	52	0.236	−0.086	0.559	.151	4,453	37	0.212	−0.294	0.718	.411	4,431	15
Cognition	Cont.	NWR (–)	−0.357	−0.633	−0.081	.011	4,573	51	−0.571	−0.898	−0.243	6.46E‐04	4,558	36	0.157	−0.351	0.664	.545	4,537	15
	Cont.	DS (–)	−0.151	−0.421	0.118	.271	4,452	53	−0.262	−0.580	0.056	.106	4,437	38	0.130	−0.375	0.635	.615	4,414	15
	Cont.	NVR (–)	−0.407	−0.682	−0.132	.004	4,190	51	−0.570	−0.897	−0.243	6.40E‐04	4,175	36	−0.014	−0.52	0.492	.957	4,154	15
	Cont.	IQ 8 (–)	−0.508	−0.778	−0.237	2.36E‐04	4,544	53	−0.617	−0.936	−0.298	1.52E‐04	4,529	38	−0.233	−0.74	0.275	.369	4,506	15

(–) = lower score is indicative of reduced performance on these metrics (for all other traits, higher scores indicate a reduced performance, or the trait has been dichotomized so that the risk group is coded as “1,” control group as “0”). Gray = binary. White = continuous. See Table 1 for expansion of abbreviations and phenotype descriptions. See Table 3 for coordinates of candidate CNVs.

^a^Binary variable (highlighted in gray), log odds ratio (logOR) given (as opposed to SMD, given for continuous variables). LCI/UCI = lower and upper bounds of 95% confidence interval. Results for split deletion/duplication results are censored in cases where there are fewer than five individuals in one of the risk groups (N1), to protect ALSPAC participants' confidentiality.

^b^N1 = numbers of individuals carrying at least one CNV for continuous variables, number of individuals carrying at least one CNV *and* in risk group for binary variables.

#### 2.5.4 Multiple testing

Correlations between phenotypes were quantified using the “mixed.cor” function of the “psych” R package (Revelle, 2015). Pearson correlations are computed for pairs of continuous variables, tetrachoric correlations for dichotomous variables, and biserial correlations for mixed pairs. After computing the correlation matrix, the effective number of tests were calculated using Nyholt's “matSpDLite” method of spectral decomposition. This method performs spectral decomposition of a correlation matrix, and then examines the ratio of observed eigenvalue variance to its possible maximum (Nyholt, 2004).

## 3 RESULTS

### 3.1 Descriptive statistics

#### 3.1.1 Correlations between phenotypes

Table 1 summarizes the descriptive data for the phenotypes studied, including sample numbers, and the numbers in the risk group (for binary/dichotomous outcomes) and means/medians (plus *SD*s and IQRs) for continuous outcomes. Several of the phenotypes were correlated. Figure 2 shows a heat map of Pearson's correlation coefficients between all outcome variables. As expected, the ASD and ADHD measures showed correlations both between and within groups. Anxiety and depression were also correlated with one another (*r* = .61), and moderately with PEs (*r*∼.35). Better performances on the cognitive measures were negatively related to the majority of the psychiatric risk traits studied, but most strongly to ASD and ADHD.

Nyholt's method of spectral decomposition calculated that there were 18 independent tests among all of the outcome variables studied (Sterne & Davey Smith, 2001).

#### 3.1.2 CNVs

Table 2 summarizes the descriptive data for CNVs. 85/6,807 (1.25%) individuals carried one or more of the 12 pathogenic CNVs (61 carried a deletion, 24 carried a duplication) summarized in Table 3, and so were excluded from the burden analyses, and descriptive data. Three pathogenic CNVs were not observed in this particular study population: the 7q11.23 duplication, the 15q11.2‐q13.1 duplication, and the 22q11.2 deletion (Kendall et al., 2016). These CNVs were all observed at frequencies of <0.01% in UK Biobank (*n* = 151,169), so it is unsurprising that we did not observe them in our study population of *n* = 6,807.

About 3,526/6,722 subjects in the burden analysis (52%) carried no rare CNVs (deletions or duplications with frequency <1%) greater than 100 kb in length. These individuals formed the reference category in the “largest carried” CNV burden analysis. The median (IQR) length of rare deletions across the genome (36 kb [0–131]) was greater than the median length of rare duplications (11 kb [0–136]). Large deletions were less common than large duplications when considering CNVs >500 kb, but >100 kb to ≤500 kb deletions were slightly more common than duplications of the same size.

### 3.2 Genome‐wide CNV burden

#### 3.2.1 Total number of affected genes

There was evidence of increased risk of higher ASD scores on the mean ASD factor (standardized mean difference [SMD] 0.021 [95%CI 0.010, 0.032], *p* = 1e‐04), with increasing numbers of genes affected by deletions. Weaker evidence of association (but concordant effect sizes) with reduced cognitive performance on the IQ measure was also observed (SMD −0.023 [95%CI −0.037, −0.009], *p* = .002).

Among the binary and dichotomized traits, the majority of associations were consistent with the null effect, although confidence intervals were wide, reflecting small sample sizes. Presence of increasing numbers of genes affected by rare deletions was associated with more problems on the coherence measure (OR 1.06 [95%CI 1.02, 1.09], *p* = 7e‐04).

When the number of genes affected by rare duplications were studied, there was weak evidence of an association with reduced coherence (OR 1.04 [95%CI 1.02, 1.06], *p* = 3e‐04), in addition to stronger evidence of more ASD symptoms, according to the mean ASD factor (SMD 0.018 [95%CI 0.011, 0.025], *p* = 3e‐07). There was also reduced performance (by about 0.01–0.02 SD units per gene affected by rare duplications) on the measures of attentional control, as well as phonological memory, working memory, and IQ (*p* < .001 for all traits).

For a graphical representation of this analysis, see Figure 3. For sensitivity analyses in which carriers of known pathogenic CNVs were retained, see Supporting Information Figure 7.

#### 3.2.2 Total length of rare CNVs

The associations for this analysis are generally similar to that of the “number of genes” analysis, above, which may be explained by the fact that the total length of rare CNV regions and the number of genes affected by rare CNVs are correlated at *r* [Spearman] = .76 (*r* [Pearson] = .64).

There was strongest evidence for an increase in total length of deletions being associated with both an increase in the mean ASD factor, and a decrease in IQ at age 8. There was evidence for similar patterns of association for the ASD factor for duplications.

See Supporting Information Figure 8 for graphical representations of the association of increasing length of total rare deletions and duplications across the genome with the phenotypes studied. These analyses excluded individuals carrying known pathogenic CNVs; Supporting Information Figure 9 shows the results of the analysis in which carriers of these known CNVs were retained.

#### 3.2.3 Largest CNV carried

Supporting Information Figures 10 and 11 shows the results of the “largest, rare CNV carried” analysis for deletions and duplications, respectively (in which individuals carrying known pathogenic CNVs were excluded). Considering the burden of multiple testing (18 independent tests), there was weak evidence of association between the presence of large, rare deletions and more problems on the coherence subscale of the CCC (OR for >500 kb to ≤1 Mb deletions: 2.82 [95%CI 1.38, 5.77], *p* = .004). There was stronger evidence for associations among the continuous outcomes: presence of rare deletions >500 kb was associated with more ASD symptoms, according to the continuous measure of ASD, and with lower IQ. The effect sizes between the >500 kb–≤1 Mb and >1 Mb deletions were broadly consistent for the ASD measure and IQ, but for other measures (selective attention, nonword repetition) there was most evidence for reduced performances with presence of >500 kb to ≤1 Mb deletions. Large duplications >1 Mb were associated with more ASD symptoms, according to the continuous measure of ASD (SMD 0.32 [95%CI 0.14, 0.51], *p* = 6e‐04).

Plots of analyses wherein individuals with any of the pathogenic CNVs were retained (as a sensitivity analysis) are available in Supporting Information Figures 12 and 13 (for the deletion and duplication analysis, respectively).

### 3.3 Candidate CNV analysis

Since CNVs associated with SCZ may be related to other neurodevelopmental traits (Kendall et al., 2016; Szatkiewicz et al., 2014), presence of CNVs robustly associated with SCZ (Kendall et al., 2016; Rees et al., 2016, 2014), was tested against the neuropsychiatric traits discussed. These results are summarized in Table 4. There were insufficient case numbers to assess the association between the pathogenic CNVs and the four psychiatric variables (anxiety, depression, ASD, and ADHD), as well as the repetitive behaviors and social communication trait.

The strongest association with candidate SCZ CNVs was observed between general cognitive ability, as measured by IQ (SMD for presence of any CNV [95%CI]: −0.508 [95%CI −0.778, −0.237], *p* = 2e‐04), with stronger evidence for deletions (SMD −0.617 [95%CI −0.936, −0.298], *p* = 2e‐04), and no evidence of association with duplications (SMD −0.233, [95%CI −0.740, 0.275], *p* = 0.369). Weaker evidence of associations were observed between the candidate deletions (but not duplications) and the mean ASD factor (SMD 0.369 [95%CI 0.124, 0.614], *p* = .003), as well as phonological memory (SMD −0.571 [95%CI −0.898, −0.243], *p* = 7e‐04), and reduced social cognition (–0.570 [95%CI −0.897, −0.243], *p* = 6e‐04).

There was no strong evidence that the SCZ candidate CNVs were associated with PEs in ALSPAC (OR 0.858 [95%CI 0.403, 1.827], *p* = .691), although confidence intervals were wide. There were insufficient cases to assess the effect of deletions and duplications separately. The low numbers of cases for the dichotomized measure of attention meant that power for these associations was low, but there was a trend toward reduced attention being associated with presence of the pathogenic CNVs.

Overall, these results provide some suggestion that the candidate deletions for SCZ are associated with reduced cognition and ASD traits, even after considering the number of comparisons made.

## 4 DISCUSSION

This study sought to analyze the relationship between the burden of rare copy number variation across the genome in relation to a wide variety of neuropsychiatric phenotypes, measured in a young (<18 years), population‐based cohort. The secondary aim was to examine the association of known SCZ CNVs with these traits.

While there have been numerous studies of rare CNVs and neuropsychiatric conditions in relation to clinically ascertained cases (such as SCZ [Grozeva et al., 2010; Kirov et al., 2009; Stone et al., 2008; Szatkiewicz et al., 2014], BPD [Grozeva et al., 2010], and autism [Ching et al., 2010; Glessner et al., 2009; Sebat et al., 2007]) and healthy controls, the relationship in population‐based samples is less well‐studied. We have prevoiusly found a relationship between the presence of rare CNVs of increasing sizes and educational attainment in ALSPAC (Männik et al., 2015), and a very recent study in a young Swedish population also confirmed associations between presence of large CNVs and neurodevelopmental problems (Martin et al., 2017). In line with this work, an association in the current analysis was observed between IQ at 8 years and the number of genes affected by rare CNVs, as well as the presence of rare deletions >500 kb. We also confirm that even in a young, unselected population, it is still possible to detect associations between genome‐wide CNVs and neuropsychiatric traits, with the association of a continuous measure of ASD also observed. Concordant with observations reported an Icelandic sample (Stefansson et al., 2014) and in UK Biobank, in which carriers of known pathogenic CNVs had impaired performance on cognitive tests (Kendall et al., 2016), we also observed associations between known SCZ CNVs and IQ, with slightly weaker evidence of associations for the continuous measure of ASD. When interpreting these results, it should be noted that there was a moderate (*r* ≅ –.3) negative correlation between the ASD/ADHD trait measures and IQ. It is therefore possible that the associations with the ASD/ADHD trait measures could be driven by larger effect sizes in lower functioning subgroups of these individuals.

With the exception of a measure of coherence (which is also correlated with IQ at −0.36), there were few clear associations observed between presence of rare CNVs and the binary traits studied. This is likely to be in part because there was less statistical power in these analyses. In addition, general cognitive ability is associated with psychiatric disorders very broadly, which may explain why the deleterious effect of CNV burden across the genome was observed most strongly for IQ (Koenen et al., 2009).

The strengths and novelty of this work include the use of an unselected population, and the use of measures that capture both clinical and sub‐threshold levels of psychopathology in a general population. However, since for traits that were primarily ascertained by maternal report (e.g., ASD traits), measurement error is more likely, and the nature of the measurement error would determine the type of any subsequent bias (i.e., if measurement error is related to CNV burden, then this could affect associations in either direction, but if it is unrelated to CNV burden, then the error would attenuate associations toward the null). Nevertheless, using questionnaire or interview‐based population‐based data is helpful, since it may be more readily attainable than clinical records of psychiatric diagnoses, which are by their nature sensitive, and require specific data linkage approval to be able to use and access them. For ASD, we had access to both clinical and research measures of ASD. Given that autism represents a spectrum, it is also possible that the genetic architecture of clinical and subclinical diagnoses may vary (as has been observed for psychosis) (Jones et al., 2016), in which case studying these measures may not only be of value as a proxy but as a means to understanding the genetic determinants of the “Broader Autism Phenotype” (Losh, Childress, Lam, & Piven, 2008). There is increasing evidence that ASD is heterogeneous, and that individual trait measures may have distinct etiologies (Happé & Ronald, 2008). However, for ADHD, it has been observed that the genetic correlation of ADHD as a disorder and ADHD traits approaches 1 (Demontis et al., 2017), and there is also genetic correlation between ASD and its associated traits (in ALSPAC [Robinson et al., 2016], and the Psychiatric Genetics Consortium [Bralten et al., 2017]). This suggests that studying ADHD/ASD traits in general populations is likely to be a powerful method that will provide useful insights into these disorders.

The lack of association between PEs and CNVs is notable because of the recent observation in ALSPAC that common genetic variation predisposing to SCZ (a polygenic risk score) also showed no strong correlation with PEs in this study (Jones et al., 2016). It has been suggested that PEs in late childhood and adolescence may be more strongly attributable to environmental factors, such as childhood trauma and substance abuse, than to genetic predisposition to SCZ (Jones et al., 2016). In addition to a lack of power, this could be another explanation for the lack of association between CNVs and PEs in this work. Other papers have also postulated that CNVs may modify the psychosis phenotype: those with CNVs may be more likely to develop SCZ, whereas those without may have a trajectory toward affective disorders with psychotic symptoms. If the PEs measured in ALSPAC are more representative of those with affective disorders than prodromal features of SCZ, this could also explain the apparent lack of a clear increased burden of rare CNVs among those with PEs (Grozeva et al., 2010).

We considered the possibility of type I and type II error in our results. The associations detected may be true positives, suggesting that the presence of rare CNVs is truly associated with cognition and ASD/ADHD traits in a general population. This fits with the results from ALSPAC published previously as part of a replication effort to study the relationship between CNVs and educational attainment in an Estonian cohort (Männik et al., 2015) as well as with the results of a large study of neurodevelopmental CNVs in relation to cognition in UK Biobank (Kendall et al., 2016). However, it is also possible that at least some of the associations are due to chance. Type I error was considered by computing the number of independent tests using a previously validated method (Nyholt, 2004). Our strongest results (the association of IQ and the composite ASD factor measure) are likely to be robust to the number of comparisons made (18 tests, equivalent *p*‐value for alpha .05: *p* = 2.78e‐03), but there is a greater possibility of false positives among the results for which there was weaker evidence. However, despite the possibility of multiple testing, the converse problem is also possible: false negatives (type II error) are likely to have been a problem for many of the binary traits, because of the relatively small numbers in the risk groups.

Another important limitation of this study is the effect of attrition: selective attrition, patterned by outcome may lead to dilution of effect sizes (Wolke et al., 2009). In ALSPAC, a polygenic risk score for SCZ has been found to be strongly associated with participant drop out, indicating that individuals at risk for SCZ may be underrepresented in ALSPAC. This may have an impact on statistical power, and may also attenuate power in genetically correlated disorders, such as depression and ADHD (Martin et al., 2016). This phenomenon might be explained by common factors lying on the causal pathway between genetic risk for SCZ, which could be responsible for attrition. Given that IQ was found to be related to rare SCZ CNVs, and educational attainment is known to be related to drop out in ALSPAC, this could be one mechanism by which study children are lost to follow up (Boyd et al., 2013). In addition, selection bias can be viewed as a collider, as participation in a study may be a common effect of the exposures and outcomes being studied. In this case, spurious correlations (“collider bias”) may be induced between the genetic exposure and outcomes under study (Martin et al., 2016; Munafo, Tilling, Taylor, Evans, & Davey Smith, 2016). This bias is likely to be worst when performing complete‐case analyses; since the analyses were not complete‐case analyses (individuals were included if they had at least one CNV passing QC, all confounding variables, plus at least one—but not necessarily all—of the outcomes), this may lessen the effect of collider bias on the results.

In conclusion, we have found some evidence of an association between the burden of rare CNVs, IQ, and psychiatric traits in a population‐based cohort of UK children, and associations between candidate CNVs for SCZ and cognition. Having shown that CNV burden and known SCZ CNVs are associated with these traits, a logical extension of this work could seek to map these associations to specific regions of the genome using a hypothesis‐free approach. The results demonstrate the utility of studying population‐based samples and nonclinical outcomes to better understand the genetic architecture of cognitive and psychiatric phenotypes.

## CONFLICT OF INTEREST

TRG reports funding from Sanofi, Biogen, and GlaxoSmithKline for projects unrelated to the work presented in this manuscript.

## Supporting information

Additional Supporting Information may be found online in the supporting information tab for this article.

Supporting Information
