# Supplementary material for: Association of copy number variation across the genome with neuropsychiatric traits in the general population
Source: Am J Med Genet B Neuropsychiatr Genet. 2018 Apr 24;177(5):489–502. doi: 10.1002/ajmg.b.32637 (PMC6099375; doi:10.1002/ajmg.b.32637)
Supplement: Supplementary file 1 — Supporting Information [file AJMG-177-489-s001.docx]

Supplement

# Supplementary Methods

Extended details of each of the phenotypes studied in this paper are given here.

## Psychiatric traits and diagnoses

### Psychotic experiences (PEs)

The Psychosis-like Symptoms (PLIKS) semi-structured interview was carried out at both 12 and 18 years in ALSPAC. The content of the interview has been described previously,^1^ but briefly, covers occurrence of hallucinations, delusions, and experiences of thought interference. At the 12-year clinic, assessors were psychology graduates trained in the Schedule for Clinical Assessment in Psychiatry (SCAN) Psychosis Section and use of the PLIKSi, and evaluated whether participants had experienced psychotic experiences (PEs) over the preceding 6 months. If a response from a participant were unclear, these were ‘rated down’, (i.e. classification of PEs was conservative). In addition, a psychiatrist was available to discuss ambiguous cases, and assessed a proportion of recorded interviews to ensure accuracy.

In this study, the primary outcome was a binary definition of suspected or definite PEs at either 12 or 18 years, versus no PEs at these ages (children were coded as having no PEs if they had no PEs recorded at 12 and/or 18 and either no PEs or a missing value at the other age).

### Anxiety and depression

Binary variables for anxiety and depression were derived using scores from the Computerised Interview Schedule — Revised (CIS-R), carried out at 18 years.^2^ This interview establishes the type and severity of neurotic symptoms, and categorises depressive episodes according to ICD-10 criteria.

Anxiety ‘cases’ were those who had the presence of symptoms for one of generalised anxiety disorder, social phobia, specific phobia, agoraphobia or panic disorder, as defined by the CIS-R. Participants were classified as having depression if they had symptoms that met the criteria for an ICD-10 diagnosis of depression (in this analysis, depression cases include mild, moderate or severe depression, and controls had no diagnosis of depression at interview).

### Autism spectrum disorder and traits (ASDs)

#### ASD diagnosis

Diagnoses of ASD have been recorded in ALSPAC via several methods, as described previously:^46^ all children given a statement of special educational needs in the Avon area were reviewed to identify those diagnosed as having ASD according to ICD-10 criteria.^47^ Maternal reports were also used to source cases, according to responses to the question (asked when children were 9 years): ‘Have you ever been told that your child has autism, Asperger’s syndrome or autistic spectrum disorder?’. Additional sources of cases included: children diagnosed by age 16, due to classification by the educational system as requiring special educational needs due to ASD; text responses to ALSPAC questionnaires relating to ASD diagnosis between 6 months to 11 years; and finally, letters from parents to the ALSPAC study director.

#### ASD traits

##### Mean of 7 ASD factors generated previously in ALSPAC

A mean of seven factors (derived from a factor analysis of 93 individual measures^5^) was also used to assess ASD.^5^ This variable was reflected and then log-transformed in order to approximate a normal distribution (where a higher score is associated with ASD). See **Supplementary Figure 1**.

##### Specific measures of ASD that predict ASD diagnosis in ALSPAC

Four ASD traits that combined optimally to form a predictive model of ASD in ALSPAC (pseudo-r^2^ reported as 0.48)^5^ were also used. The coherence subscale of the Children's Communication Checklist (CCC), was scored at 9 years,^6^ and includes questions such as whether the child could explain the rules of a simple game to a younger child. The Social and Communication Disorders Checklist (SCDC, 91 months)^7^ includes questions such as whether the child was able to realise if they had offended people, and about whether they responded to instructions. Another measure explained the presence of repetitive behaviours (RB) at 69 months).^8^ Finally, the sociability subscale of the Emotionality Activity and Sociability (EAS) included measures of whether the child enjoyed the company of people at age 38 months.^9^

The RB and SCDC, CCC measures were highly skewed and were therefore dichotomised, defining 10% of the sample for each group defined as the ‘risk’ group for ASD traits. The SCDC has previously been diagnosed using a cut-off of 9,^10^ and in this paper, the ‘10%’ rule applied to dichotomise skewed traits came up with a similar cut-off of 8. No other precedents for cut-offs were found for other ASD traits. All traits were based on responses to questionnaires filled out by mothers. The EAS was approximately normally distributed, and thus was analysed continuously, after reflection so that a higher score was associated with increased risk of ASD. See **Supplementary Figure 2**.

### Attention-deficit hyperactivity disorder, and associated traits

#### ADHD diagnosis

ADHD diagnoses are recorded in ALSPAC according to DSM-IV criteria, and based on the Development and Well-Being Assessment (DAWBA) assessment at 91 months. This diagnosis variable was a composite of children with combined, inattentive, or hyperactive-impulsive ADHD diagnoses. Children with pervasive developmental disorders were excluded.

#### Hyperactivity

Hyperactivity was assessed using the hyperactivity score from the Strengths and Difficulties Questionnaire (SDQ).^11^ This measure was derived from the results of a maternal questionnaire administered when children were 81 months old, and provides a quantitative measure of ADHD that can also be used to generate a categorical definition.[55]. Since the measure was zero-inflated in the participants included in this study, this variable was analysed as a dichotomous variable, defining a 10% risk group. This was equivalent to using a cut-off of 7, as previously suggested.^12^ See **Supplementary Figure 3**.

#### Inhibition and impulse control

Inhibition and impulsivity were measured using the ‘Stop-Signal Inhibition’ task.^13–15^ The task begins with a familiarisation step. A small smiley face was presented in the centre of a computer screen, and an ‘X’ or an ‘O’ would then appear: children were asked to click on a corresponding button as quickly as possible, according to the symbol that appeared. Thirty such trials were completed, and mean reaction times were computed.

In the test phase, faces were still presented, but sometimes, a bleep was sounded (the ‘stop signal’) after presentation of the ‘X’ or ‘O’ (the ‘go signal’). In this case, the child was requested to refrain from clicking the corresponding button, as in the familiarisation phase. Bleeps were presented randomly at either 150 milliseconds (ms) or 250ms intervals before the child’s mean reaction time.

If children had hearing impairments, a visual signal was provided. For those children with use of only one hand, a one-handed stimulus box was used.

The number of trials correct at the 150ms and 250ms were used in this analysis. Both variables were dichotomised (into 10% risk groups). See **Supplementary Figure 4**.

#### Attention

Two measures of attention, capturing selective attention and attentional control were used (both measured at 8 years), and were assessed using subscales of the Test of Everyday Attention for Children ‘TEACh’ task.^16^ Selective attention was measured using the ‘Sky Search’ task, in which children were given pairs of both identical and non-identical spaceships, and were required to circle identical pairs as quickly as possible, after an adjustment for motor speed. The ‘Opposite Worlds’ task measured attentional control: a list of 24 items (consisting of either the number 1 or 2), was presented to the child. The control (‘Same World’) trial involved the child reading out the numbers as they appeared, as quickly as possible. In the test (‘Opposite World’) trial, the child had to read ‘one’ on seeing a ‘2’, and ‘two’ on seeing a ‘1’. The test trial was repeated twice, and the mean time of both trials was taken as the variable for this analysis. Both measures were logged as this approximated a better normal distribution. Three observations with values <0 were dropped, to facilitate logging of the selective attention variable. For both attentional measures, a higher score indicates a less favourable score (as the measures were based on response times). See **Supplementary Figure 5**.

## Neurocognitive traits

See **Supplementary Figure 6**.

### Memory

In addition, specific measures of memory were analysed using the digit span aspect of the WISC, administered at 8 years.^17^ The digit span task measures working memory, and requires memorising and repeating back lists of digits of varying lengths. The total raw score on this task was used. The non-word repetition task measures phonological memory, and was an adaptation of a previously published task:^18^ children were asked to repeat 12 nonsensical (yet conforming to English language-like sound combinations) of three, four or five syllables. Words were delivered via an audio cassette. The total number of correct tasks was used in this analysis. Therefore, for both of these measures, a higher score is favourable. Both measures were normally distributed.

### Social cognition

A measure of social cognition (non-verbal recognition) was measured using the number of errors made on the Diagnostic Analysis of Non-Verbal Accuracy (DANVA) face recognition task.^19^ This variable has been used previously in studies of ALSPAC psychiatric traits.^20,21^ This task involves the child being asked to assign emotions (‘Happy’, ‘Sad’, ‘Angry’, ‘Fearful’) to a sequence of 24 photographs of faces, each face being displayed (either on a computer or manually) for approximately two seconds. In this analysis, the number of errors made was used as the outcome variable, after reflecting (see **Supplementary Figure 4**) the variable, so that a higher score indicated a favourable performance (in line with the other cognitive variables studied).

### Intelligence Quotient (IQ)

Intelligence quotient (IQ) was measured at 8 years by the Wechsler Intelligence Scale for Children (WISC).^17^ A higher score indicates a higher IQ. IQ was normally distributed.

# Supplementary Notes

## Supplementary Note 1

Coordinates for telomeric regions (NB: NCBI build 36 / hg18)

chr1:1-500000

chr1:246749719-247249719

chr2:1-500000

chr2:242451149-242951149

chr3:1-500000

chr3:199001827-199501827

chr4:1-500000

chr4:190773063-191273063

chr5:1-500000

chr5:180357866-180857866

chr6:1-500000

chr6:170399992-170899992

chr7:1-500000

chr7:158321424-158821424

chr8:1-500000

chr8:145774826-146274826

chr9:1-500000

chr9:139773252-140273252

chr10:1-500000

chr10:134874737-135374737

chr11:1-500000

chr11:133952384-134452384

chr12:1-500000

chr12:131849534-132349534

chr13:1-500000

chr13:113642980-114142980

chr14:1-500000

chr14:105868585-106368585

chr15:1-500000

chr15:99838915-100338915

chr16:1-500000

chr16:88327254-88827254

chr17:1-500000

chr17:78274742-78774742

chr18:1-500000

chr18:75617153-76117153

chr19:1-500000

chr19:63311651-63811651

chr20:1-500000

chr20:61935964-62435964

chr21:1-500000

chr21:46444323-46944323

chr22:1-500000

chr22:49191432-49691432

## Supplementary Note 2

Coordinates for centromeric regions (NB: NCBI build 36 / hg18)

chr1:121100001-128000000

chr2:91000001-95700000

chr3:89400001-93200000

chr4:48700001-52400000

chr5:45800001-50500000

chr6:58400001-63400000

chr7:57400001-61100000

chr8:43200001-48100000

chr9:46700001-60300000

chr10:38800001-42100000

chr11:51400001-56400000

chr12:33200001-36500000

chr13:13500001-18400000

chr14:13600001-19100000

chr15:14100001-18400000

chr16:34400001-40700000

chr17:22100001-23200000

chr18:15400001-17300000

chr19:26700001-30200000

chr20:25700001-28400000

chr21:10000001-13200000

chr22:9600001-16300000

## **Supplementary Note 3**

Coordinates for immunoglobulin regions (NB: NCBI build 36 / hg18)

chr22:20715572-21595082

chr14:105065301-106352275

chr2:88937989-89411302

chr14:21159897-22090937

# Supplementary Figures

## Supplementary Figure 1


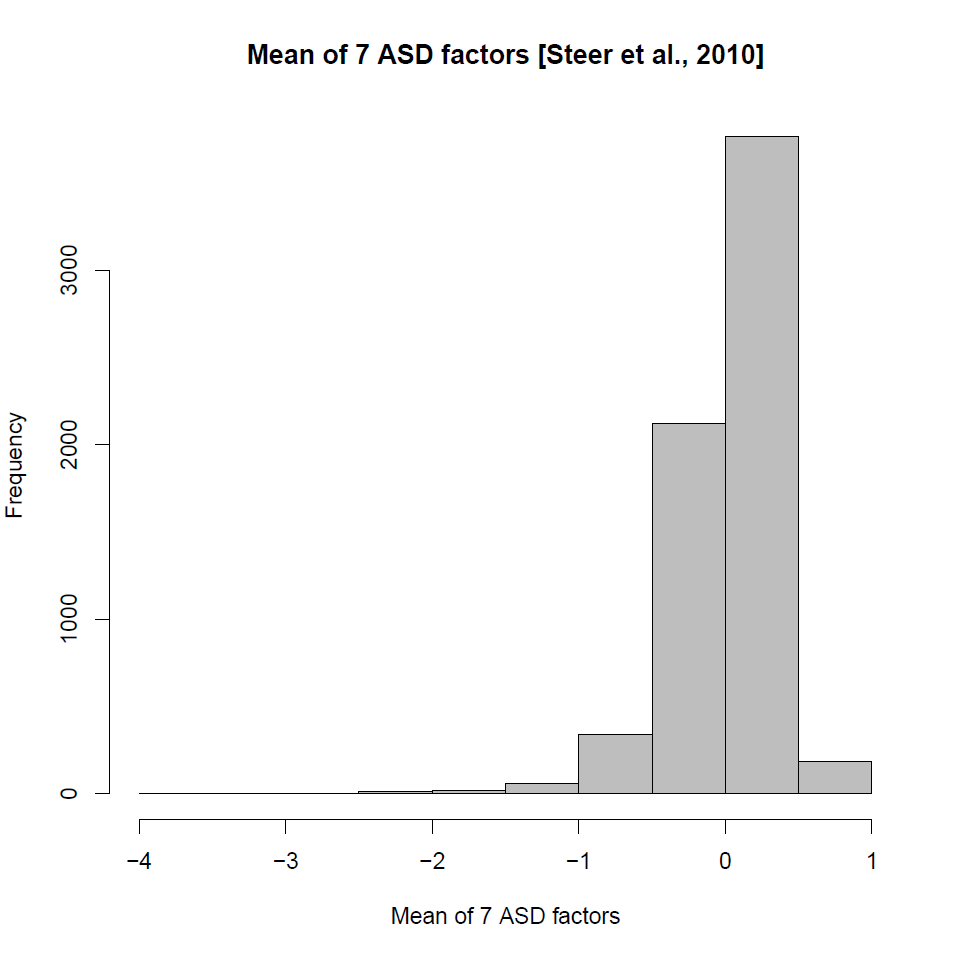

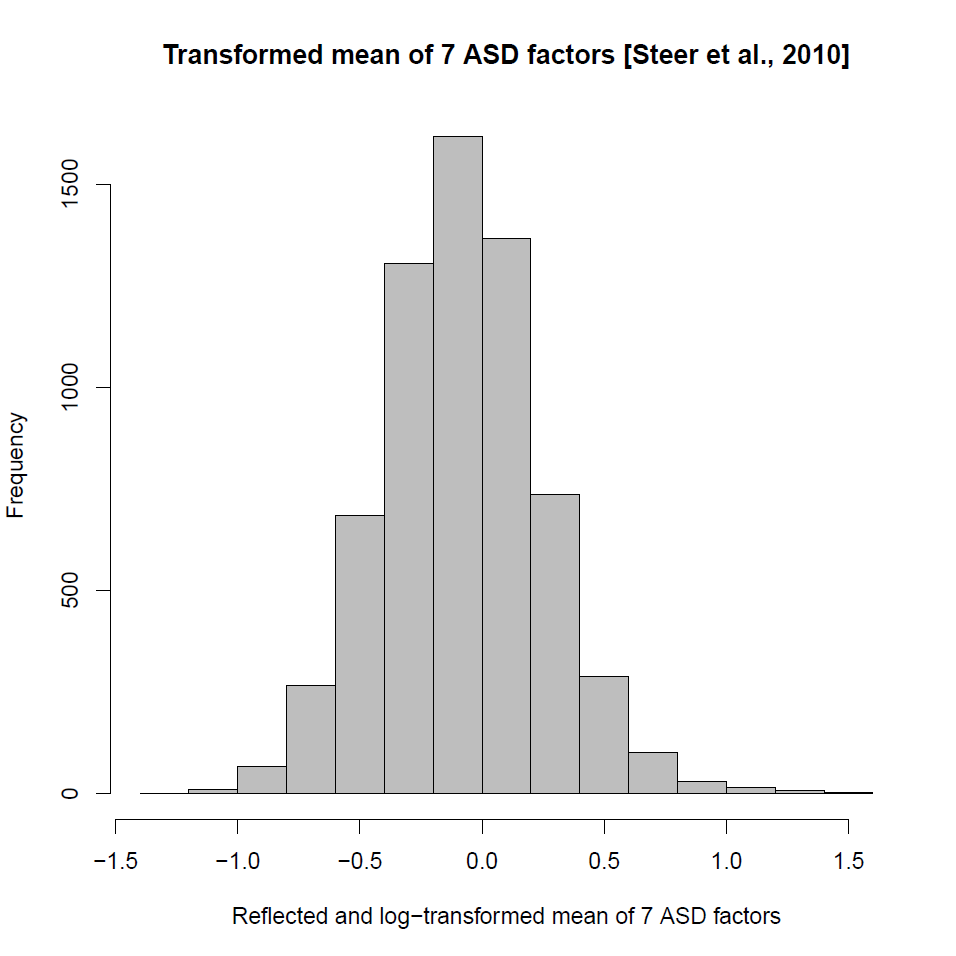


Histograms of raw (left) and transformed (right) mean of 7 ASD factors (from Steer et al., 2010)

## Supplementary Figure 2


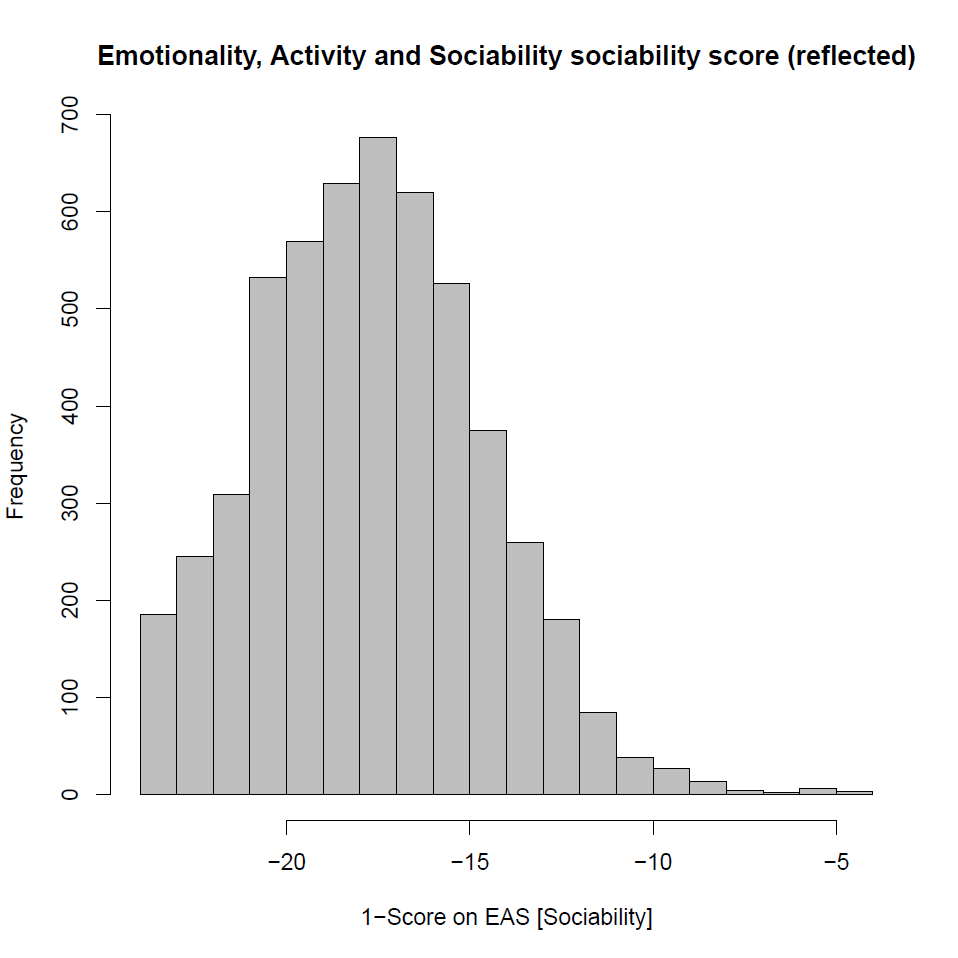

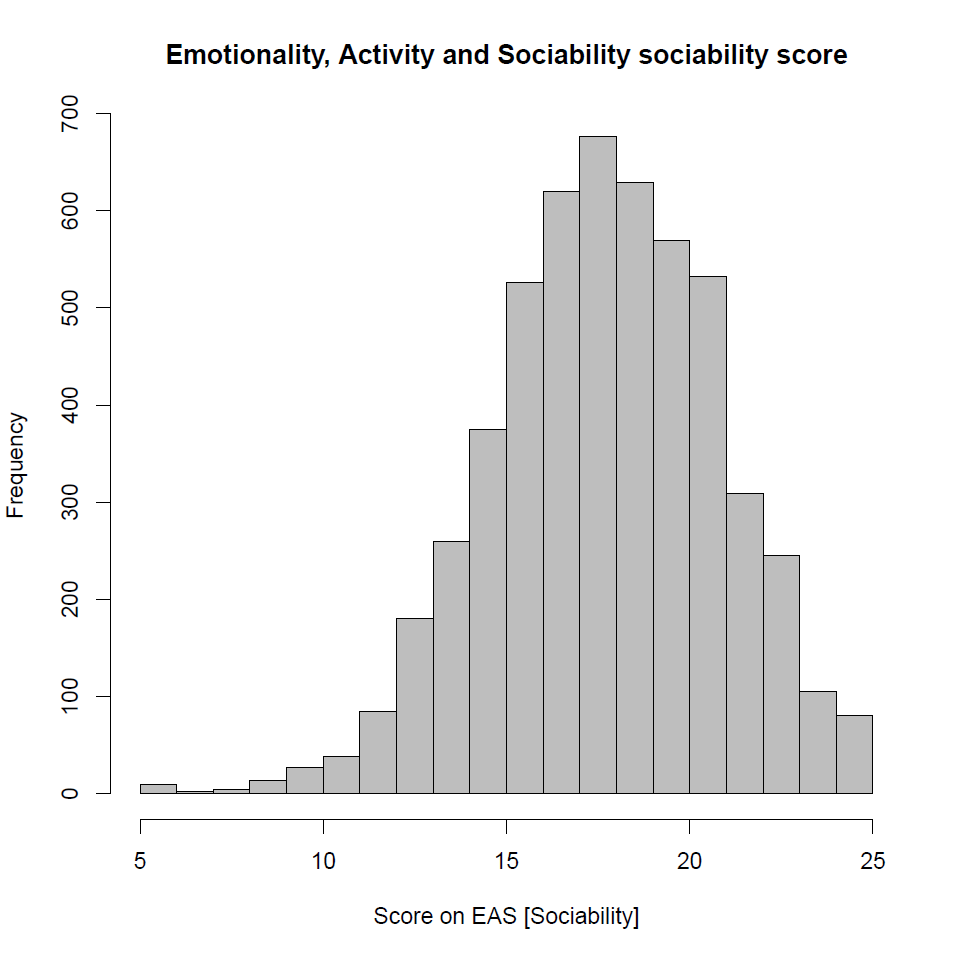


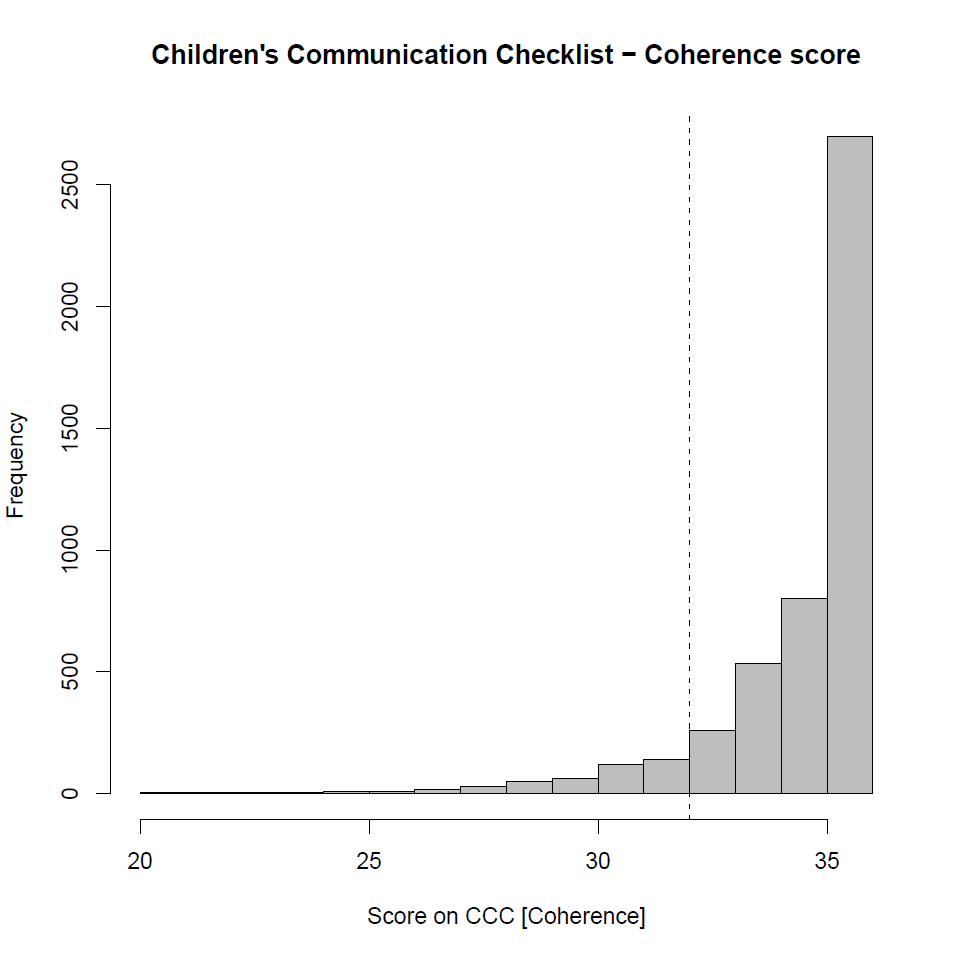

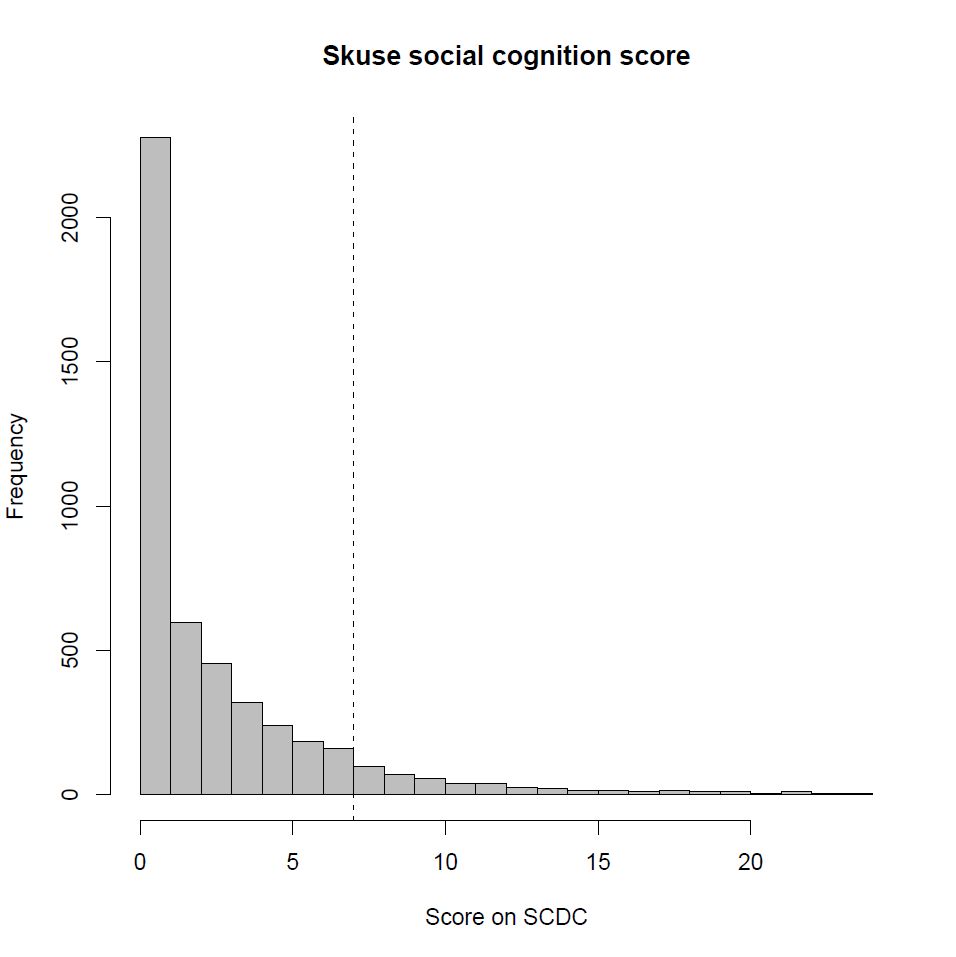


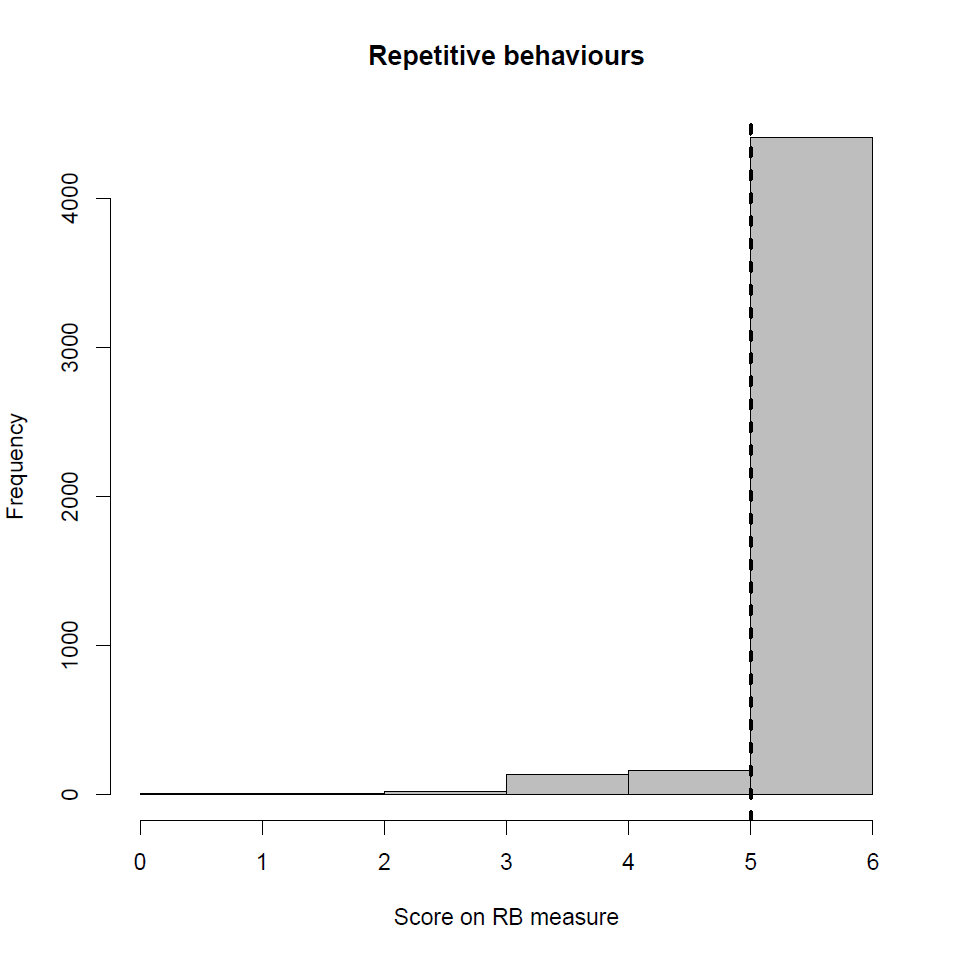
Histograms of four ASD traits in ALSPAC. The EAS (top row) was first reflected so that a higher score was consistent with ASD risk, and then analysed continuously (after standardisation). The other traits were skewed, and so were transformed, defining an approximate 10% risk group for each. Dotted lines indicate dichotomisation thresholds. Higher scores indicate the risk groups for the Skuse social cognition score (SCDC, middle right). For the repetitive behaviour measure (bottom), 93% of participants scored the lowest possible (more neurotypical) score, meaning that the proportion in the ‘risk’ group was only 6.9%.

## Supplementary Figure 3


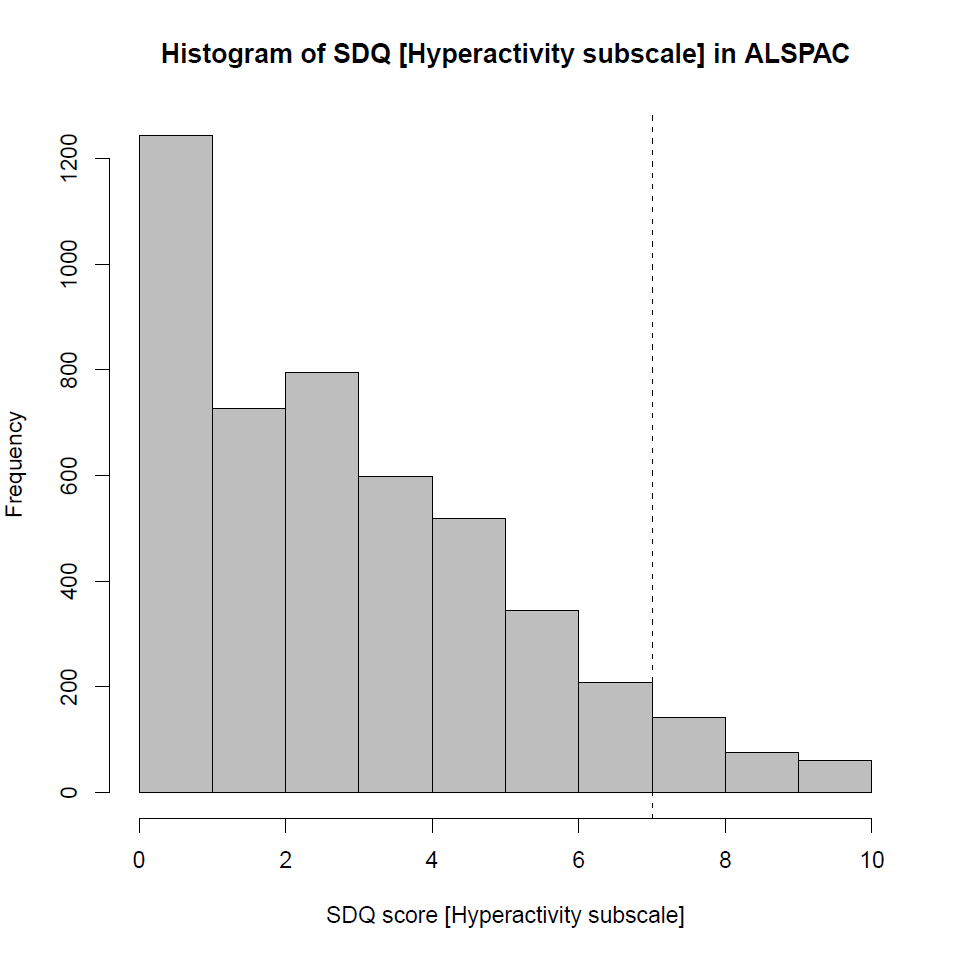


Histogram of SDQ [score out of 10 on Hyperactivity scale] in ALSPAC (dotted line indicates threshold for dichotomisation, 7)

## Supplementary Figure 4


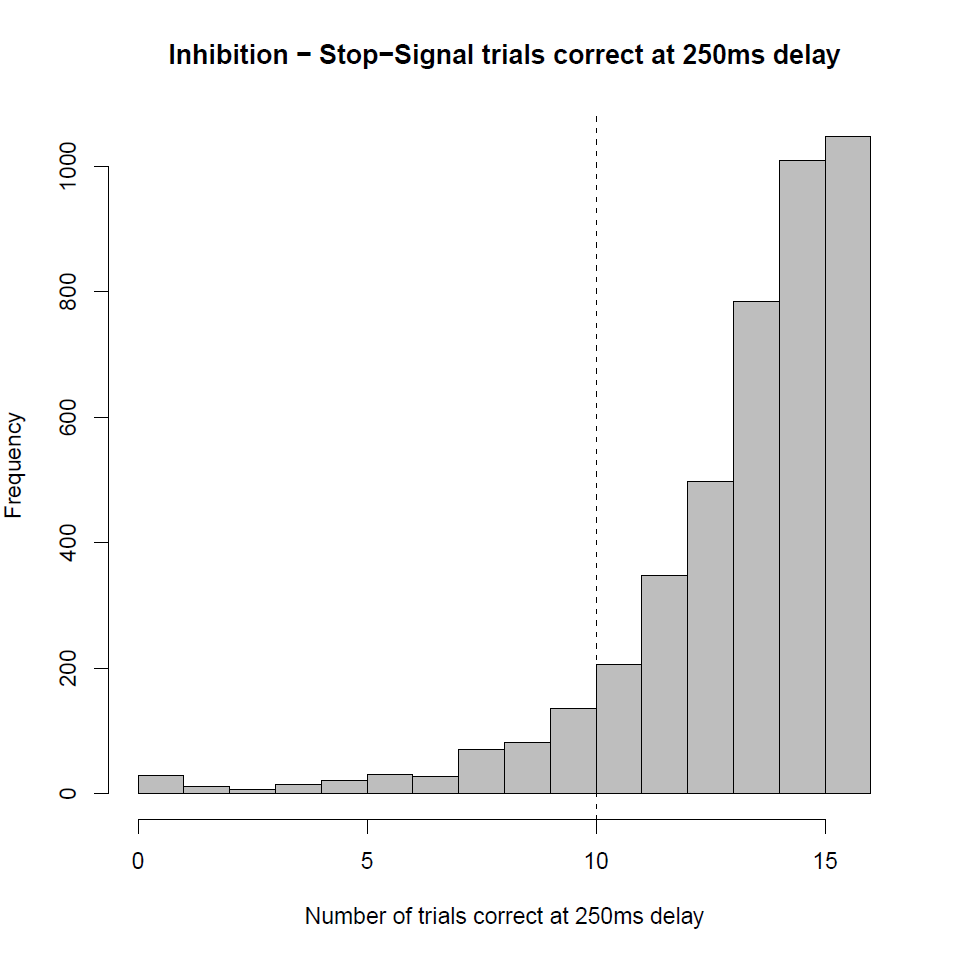

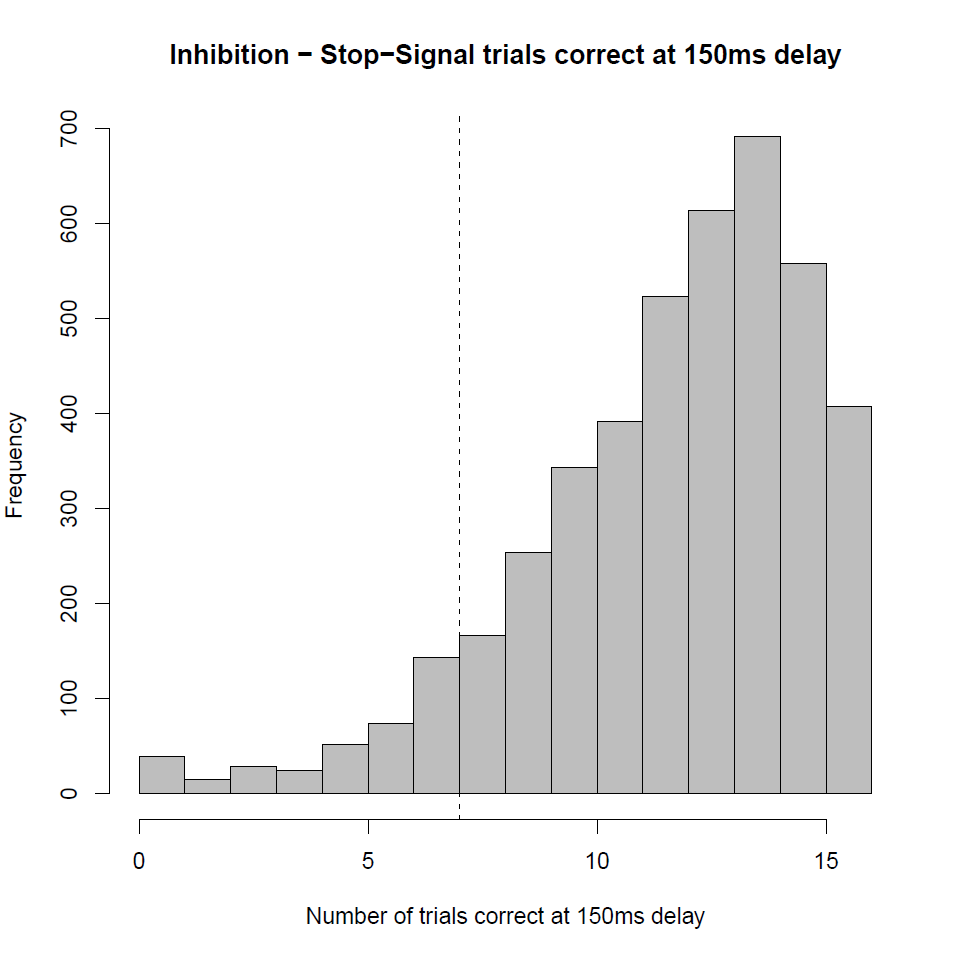


Number of trials correct in two stages (250ms [left] and 150ms [right] delay) of the Stop-Signal Inhibition task in ALSPAC (dotted line indicates dichotomisation threshold).

##
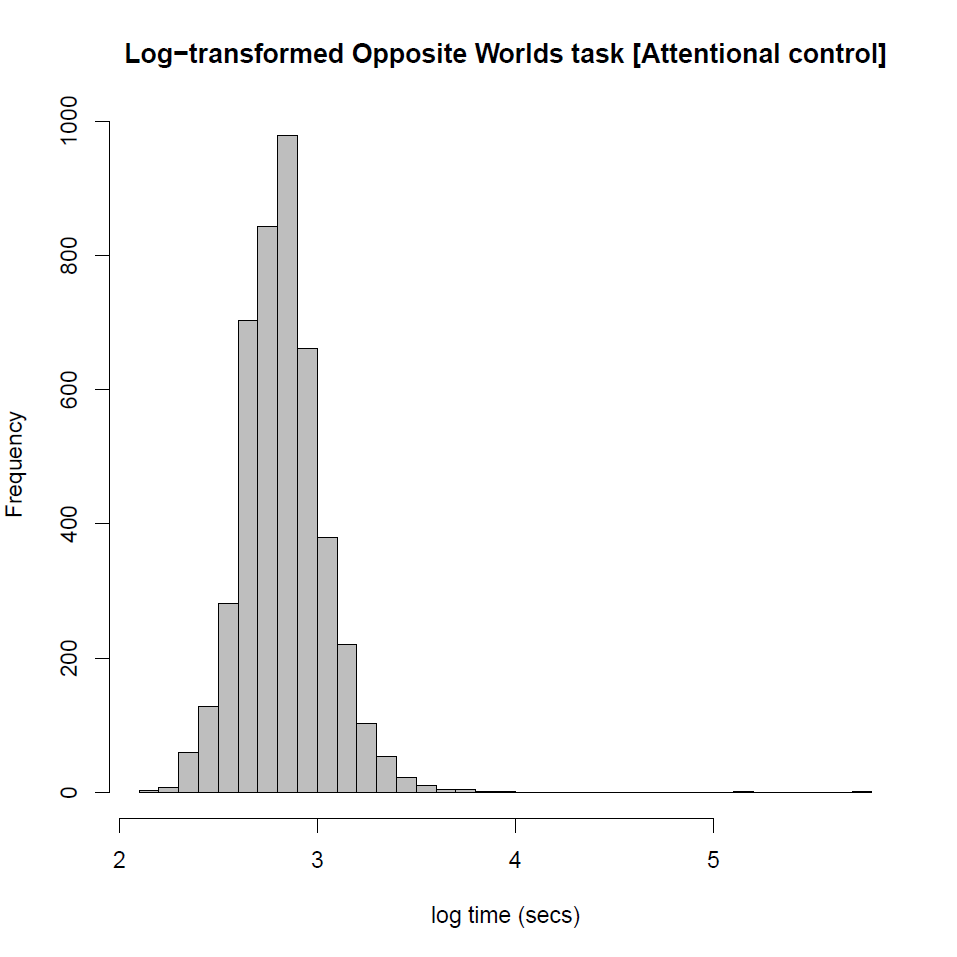

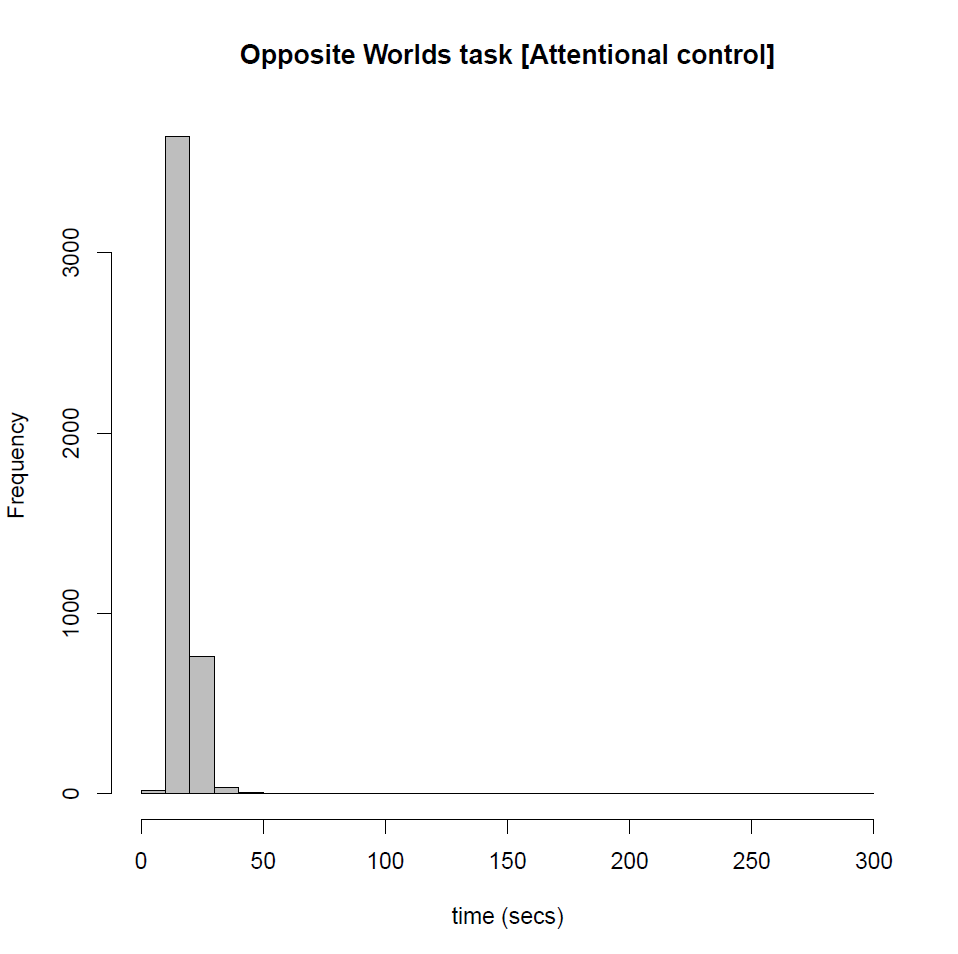

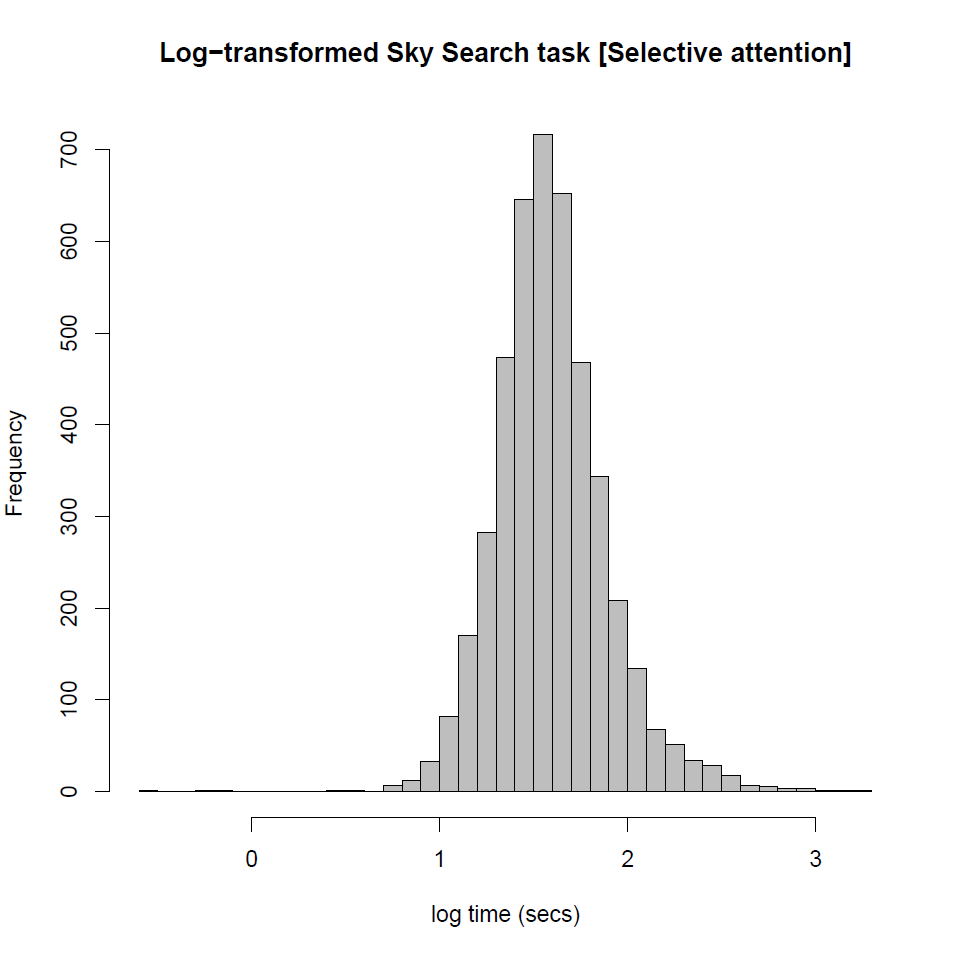

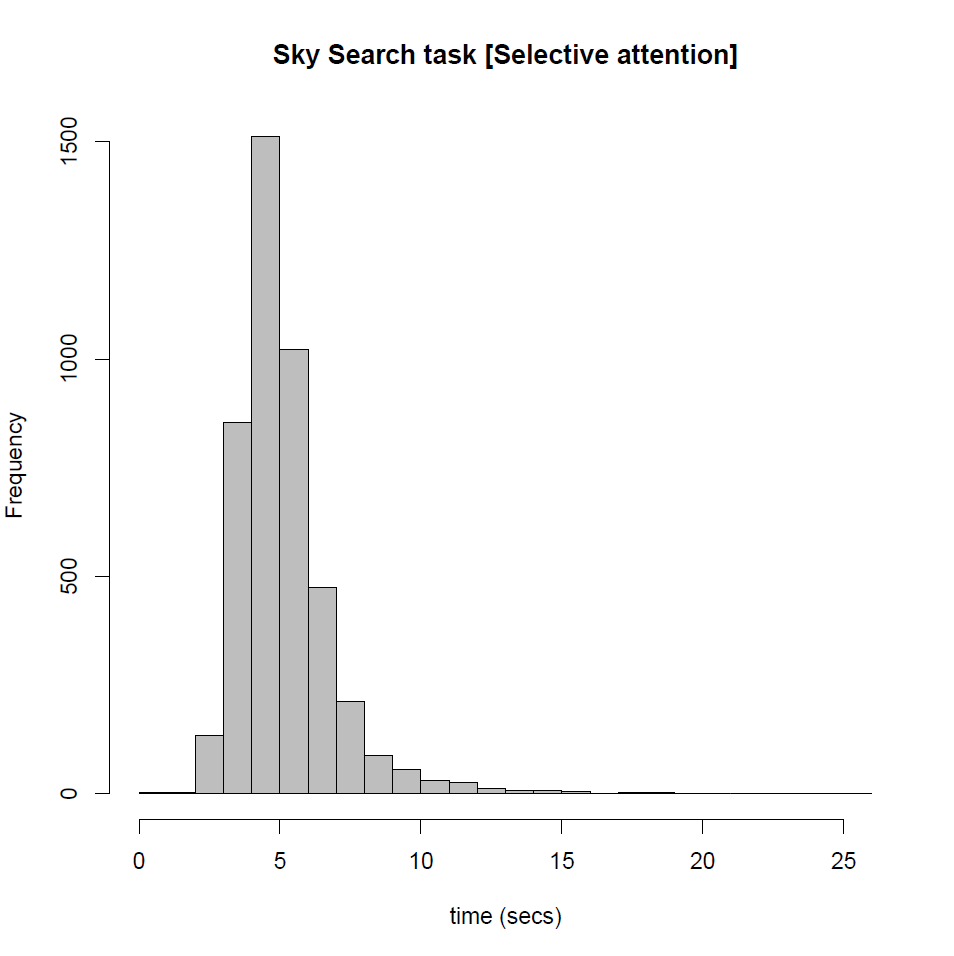
Supplementary Figure 5

Histograms of attention variables in ALSPAC. Both variables were skewed (left plots), and so were log-transformed (right plots), to better approximate normality, before standardisation and analysis.

##
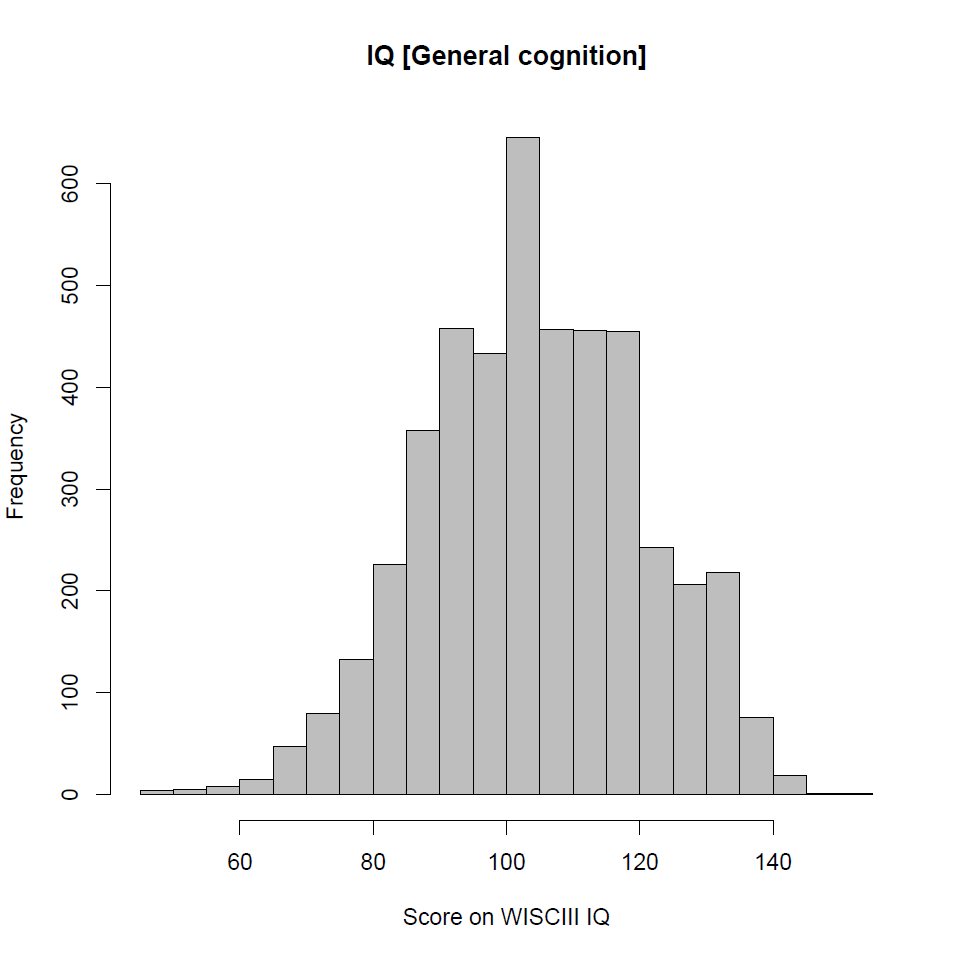

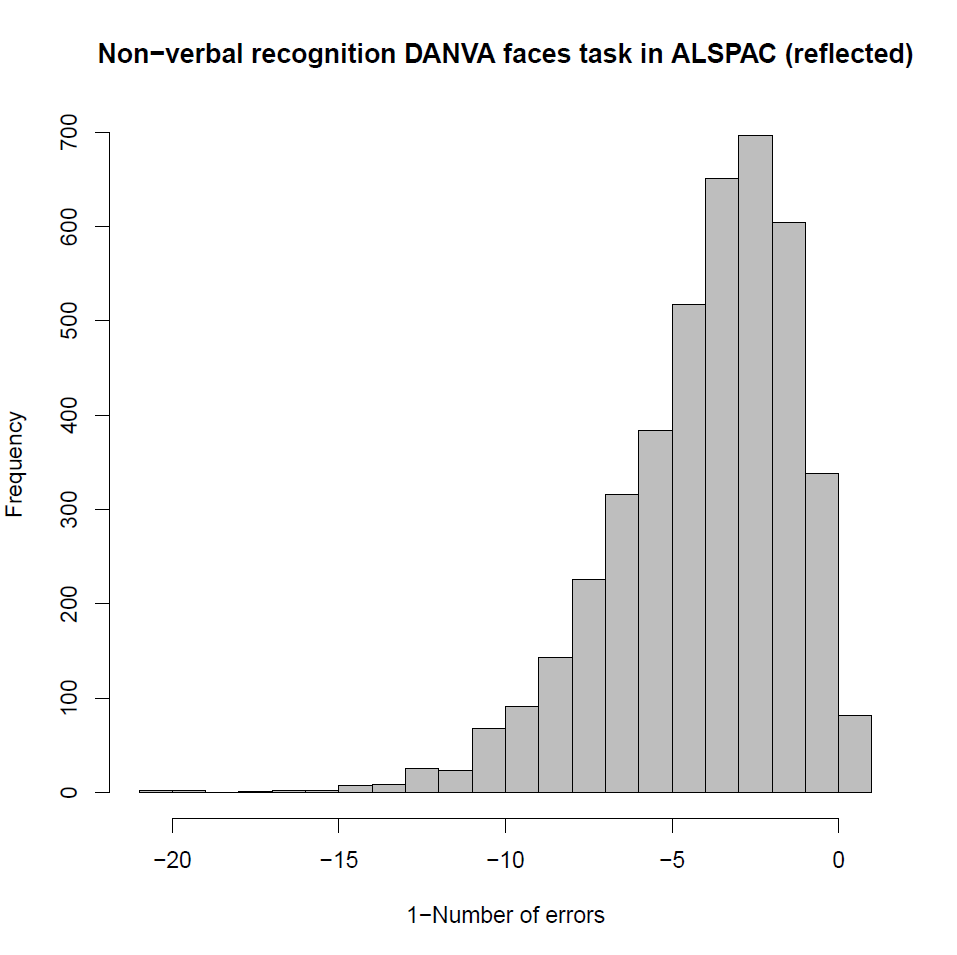

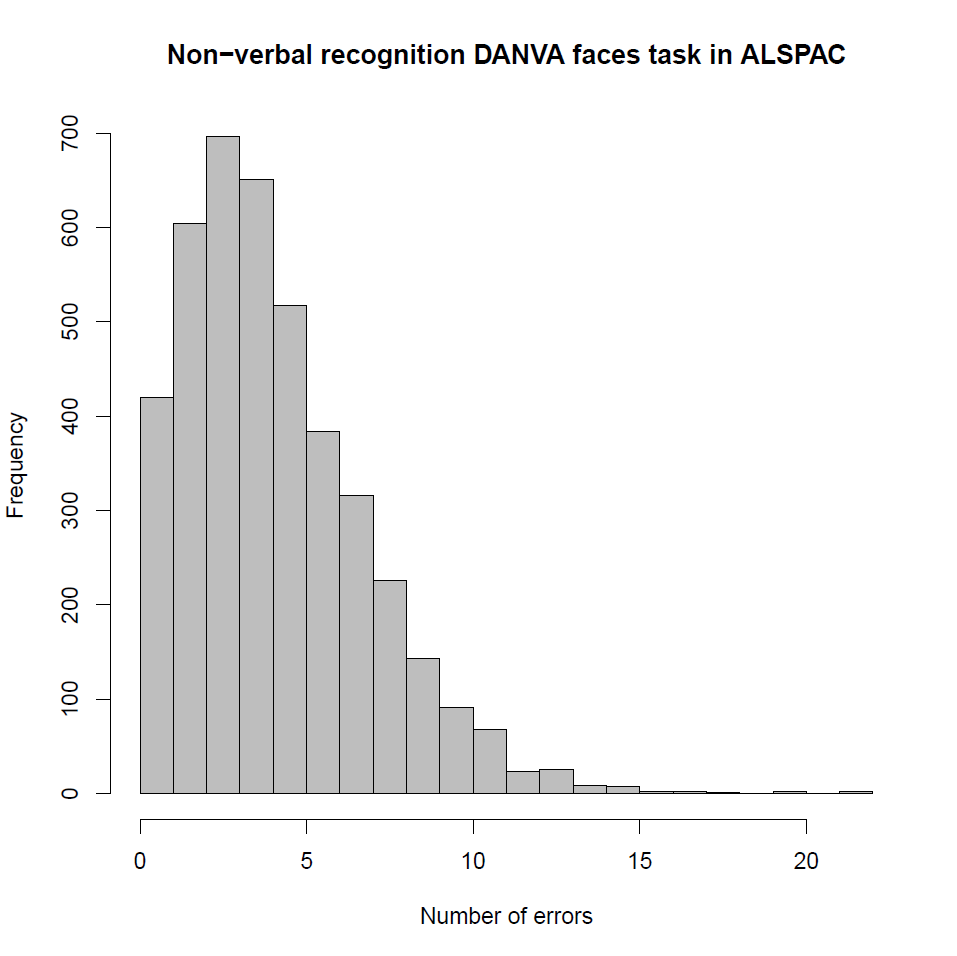

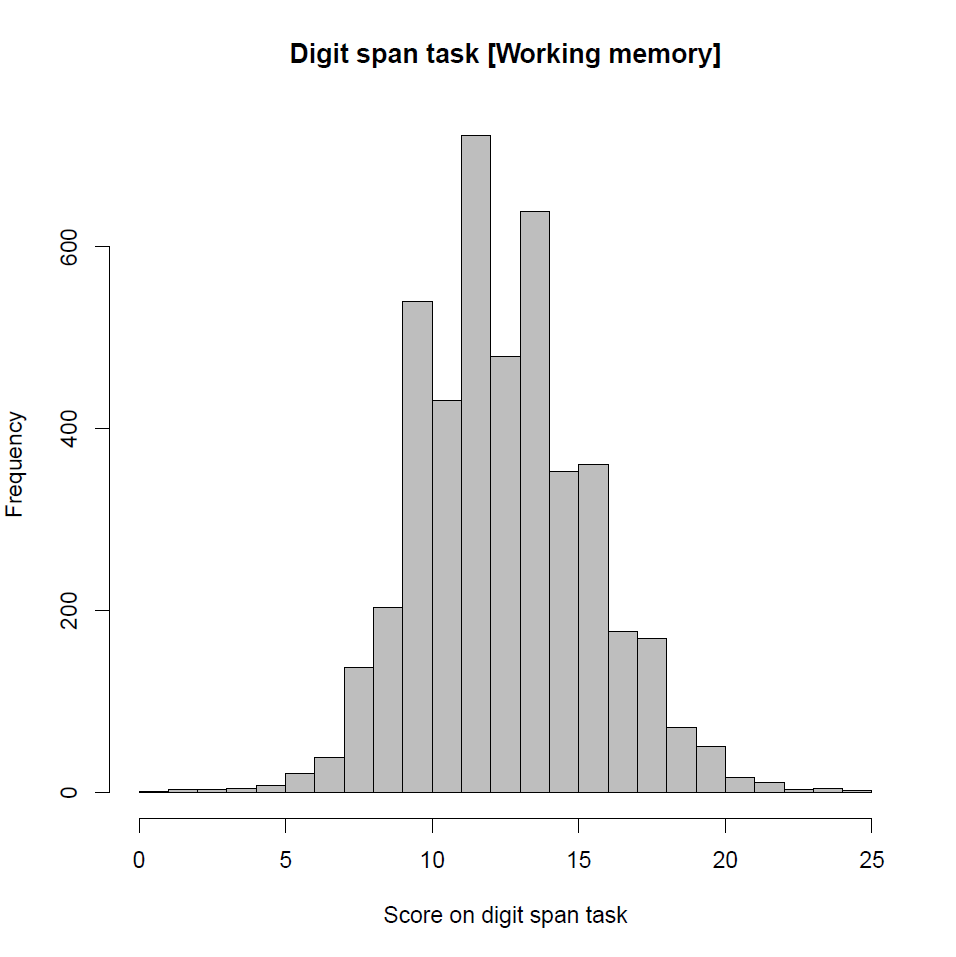

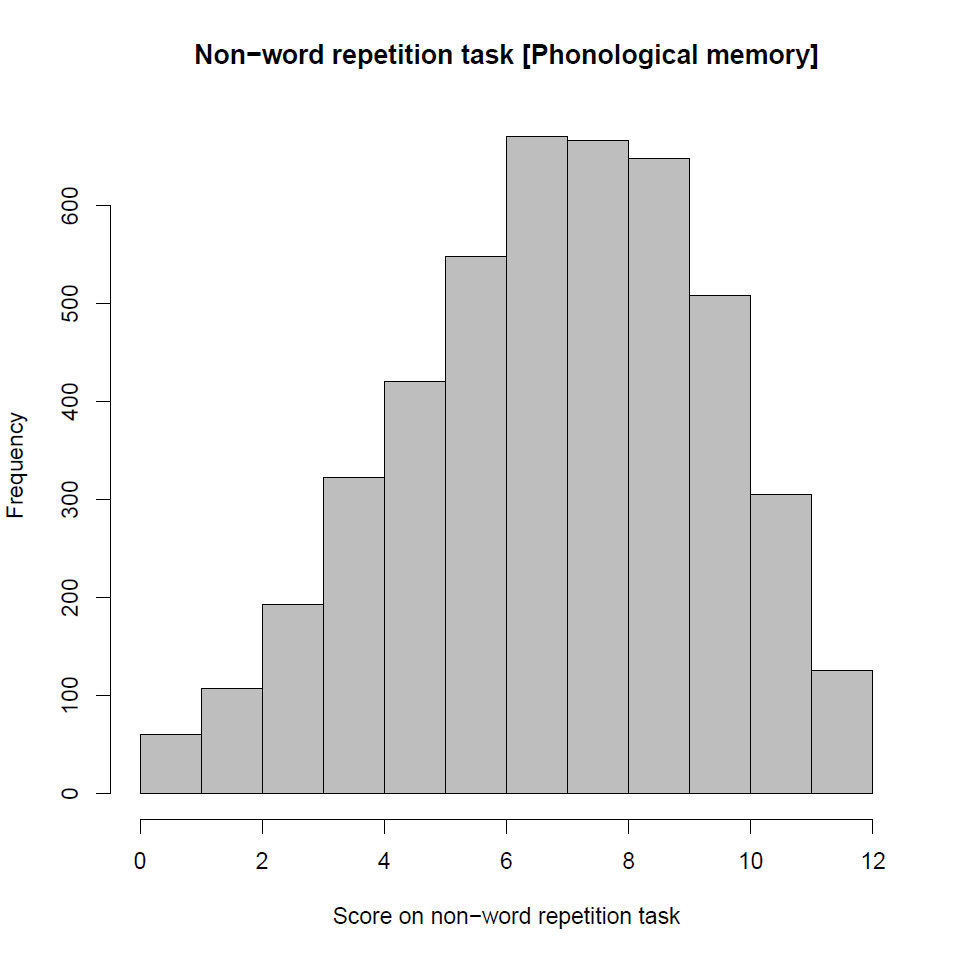
Supplementary Figure 6

Top: approximately normally distributed variables of memory. Middle: Histograms of social cognition [non-verbal recognition, number of errors out of 24 on DANVA faces task] in ALSPAC. The raw variable (left) was reflected (right), so that a higher score indicates a favourable performance (in line with other cognitive variables studied), and then standardised. Bottom: IQ was normally distributed.

All variables were standardised before analysis.

## Supplementary Figure 7

Analysis of number of genes affected by rare CNVs, separately by deletions (top) and duplications (bottom). **NB: in this analysis, individuals carrying known pathogenic CNVs are retained.**
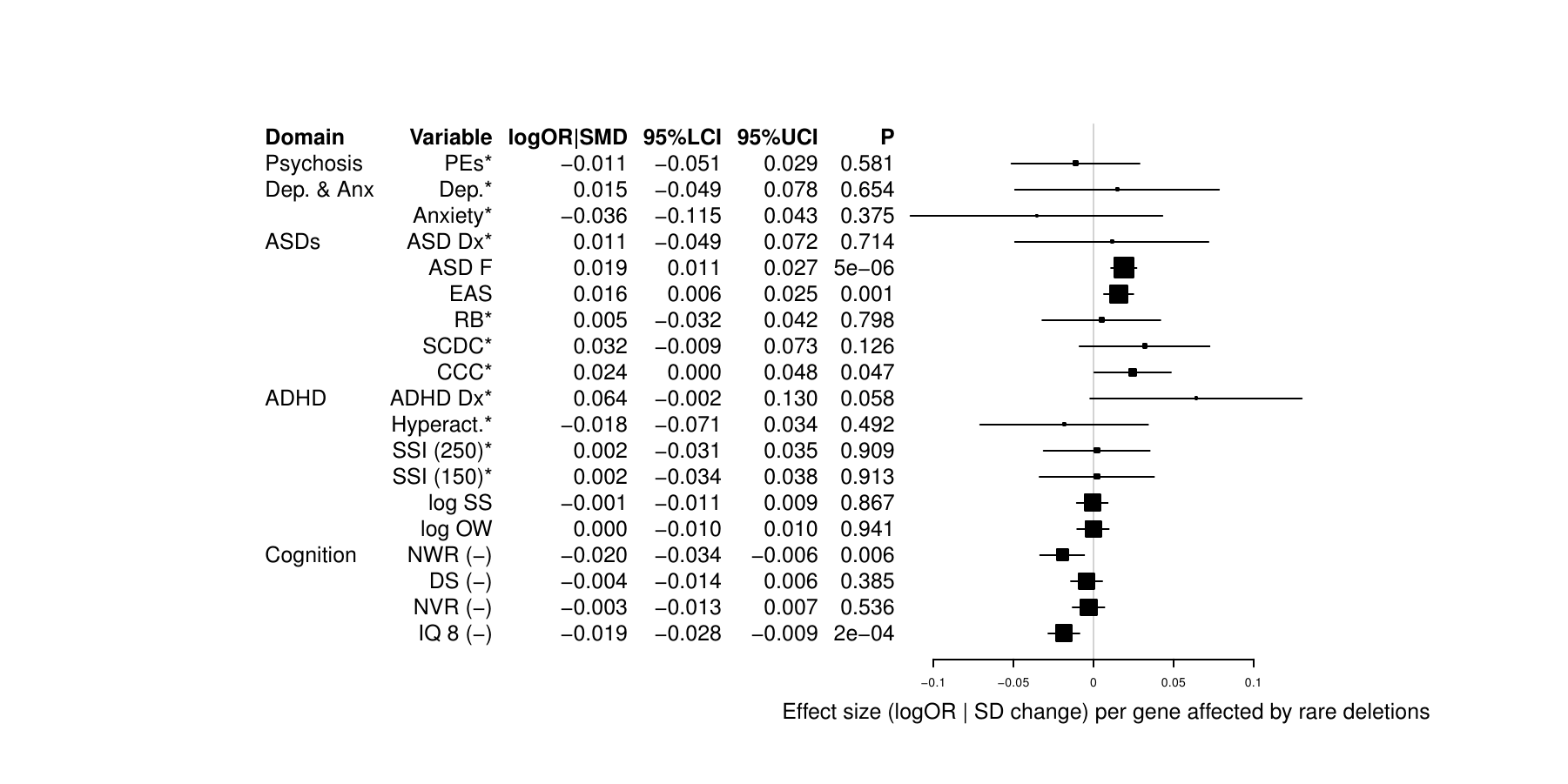

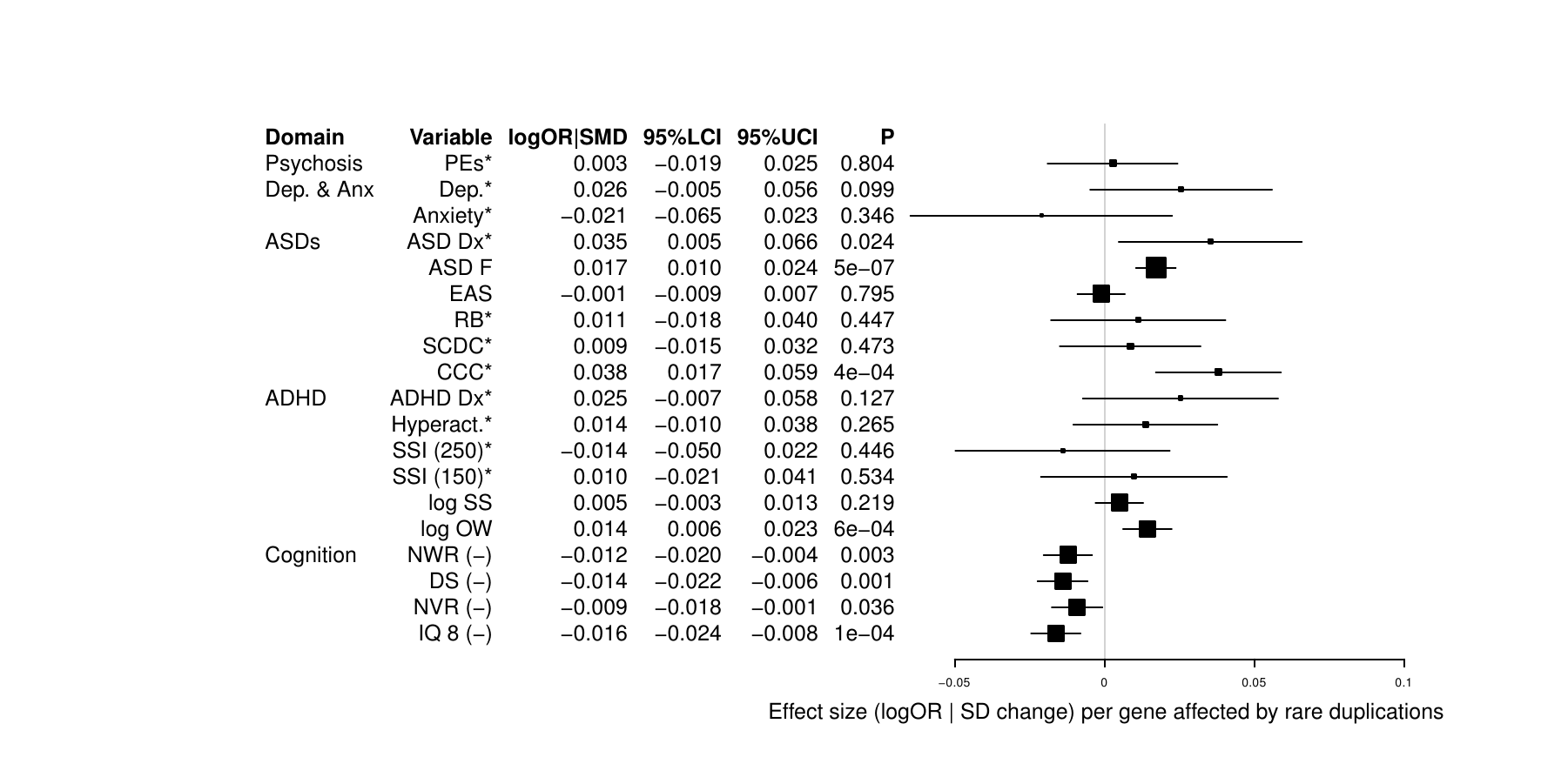


For expansion of trait abbreviations, see Table 1. Other abbreviations: logOR|SMD =effect size (logOR for binary traits [denoted with a *], SD change for continuous traits); LCI/UCI=lower and upper bounds of 95% confidence interval; (-)=lower score is indicative of reduced performance on these metrics (for all other traits, higher scores indicate a reduced performance, or the trait has been dichotomised so that the risk group is coded as ‘1’, control group as ‘0’).

## Supplementary Figure 8

Analysis of total length of rare CNVs carried, separately by deletions (top) and duplications (bottom).


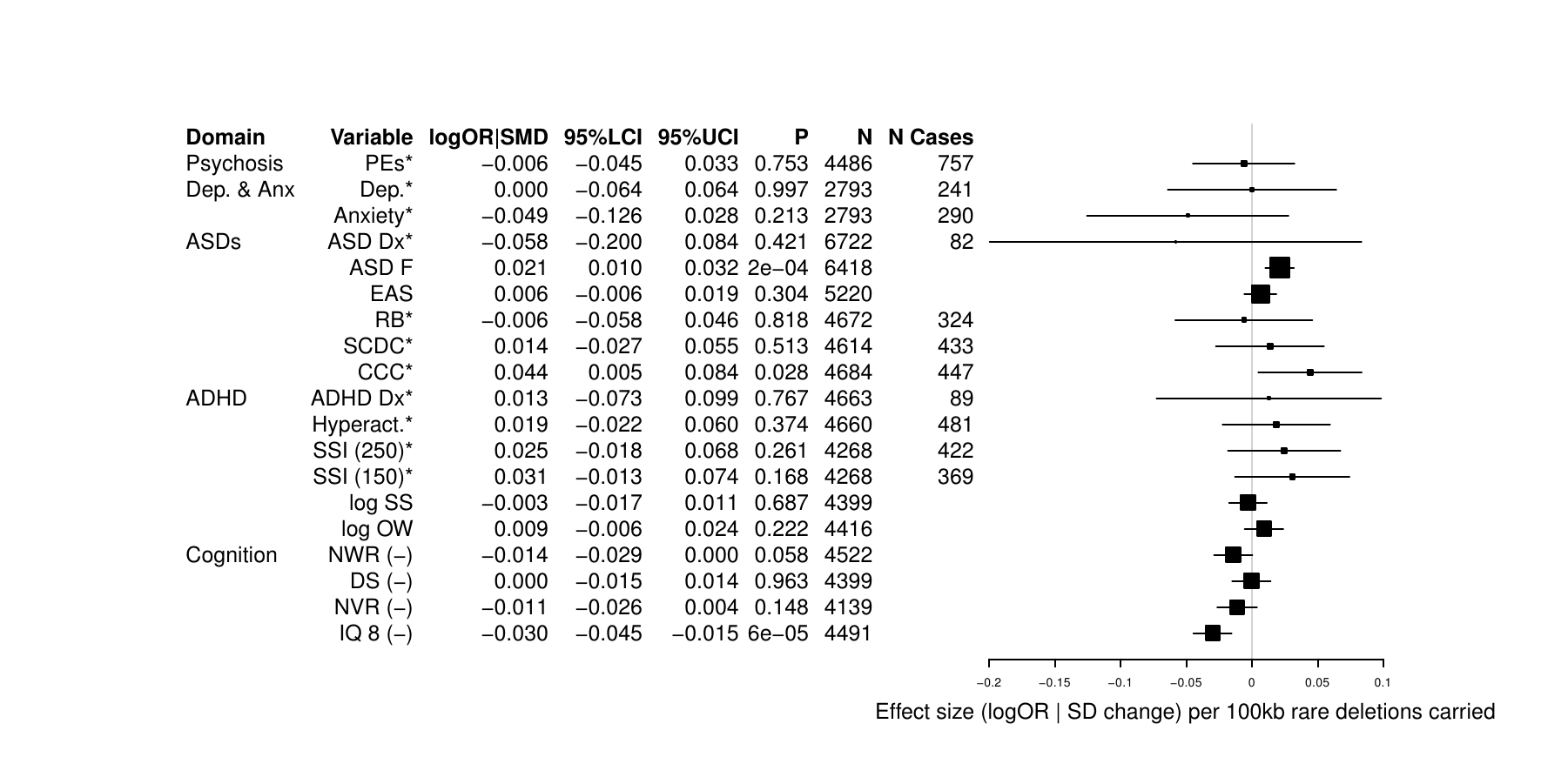

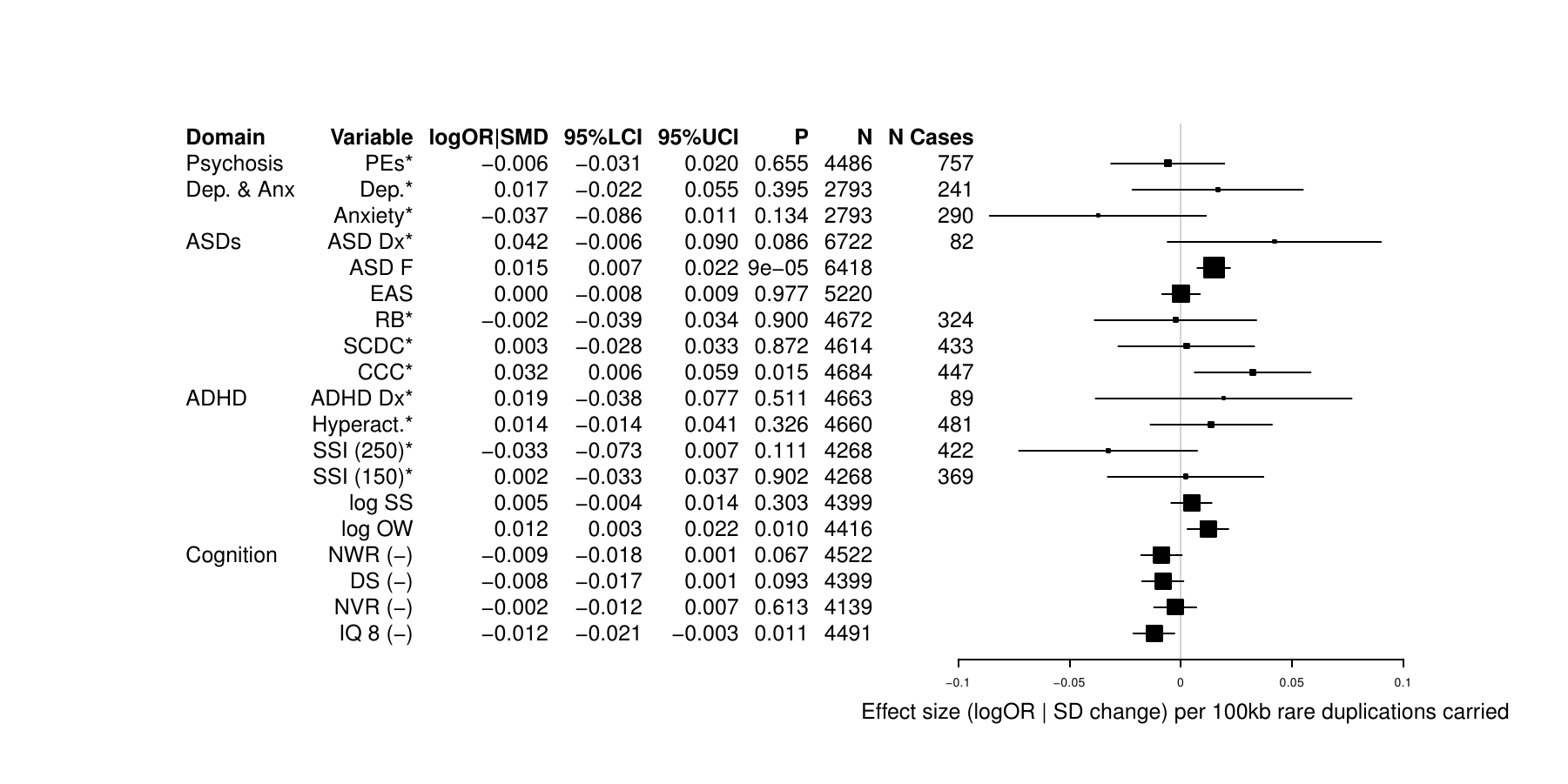


For expansion of trait abbreviations, see Table 1. Other abbreviations: logOR|SMD=effect size (logOR for binary traits [denoted with a *], SD change for continuous traits); LCI/UCI=lower and upper bounds of 95% confidence interval; N=total sample size; N cases=numbers of individuals carrying at least one CNV for continuous variables, number of individuals carrying at least one CNV and in risk group for binary variables. (-)=lower score is indicative of reduced performance on these metrics (for all other traits, higher scores indicate a reduced performance, or the trait has been dichotomised so that the risk group is coded as ‘1’, control group as ‘0’).

## Supplementary Figure 9

Sensitivity analysis of total length of rare CNVs carried, separately by deletions (top) and duplications (bottom). **NB: in this analysis, individuals carrying known pathogenic CNVs are retained.**


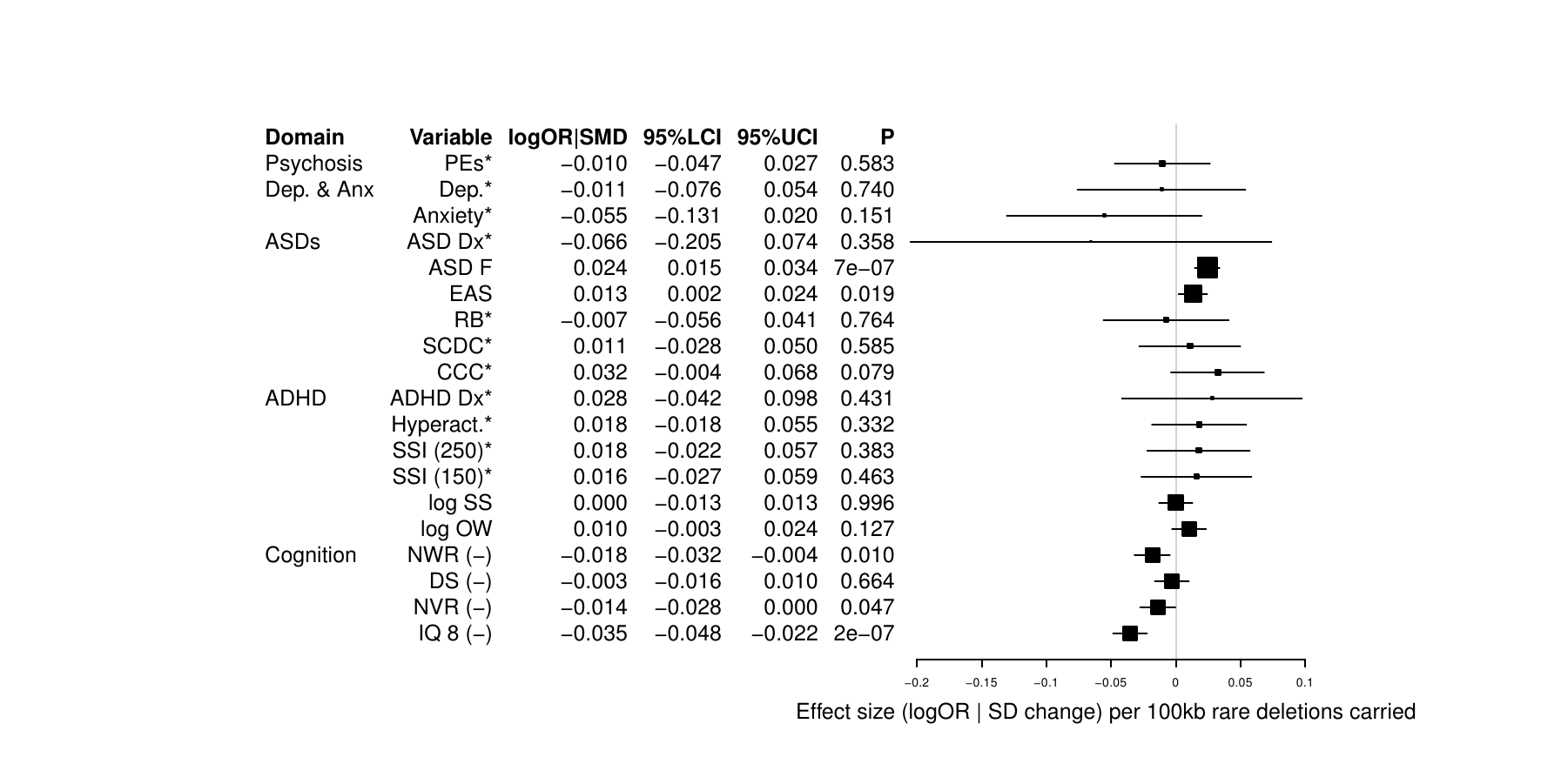

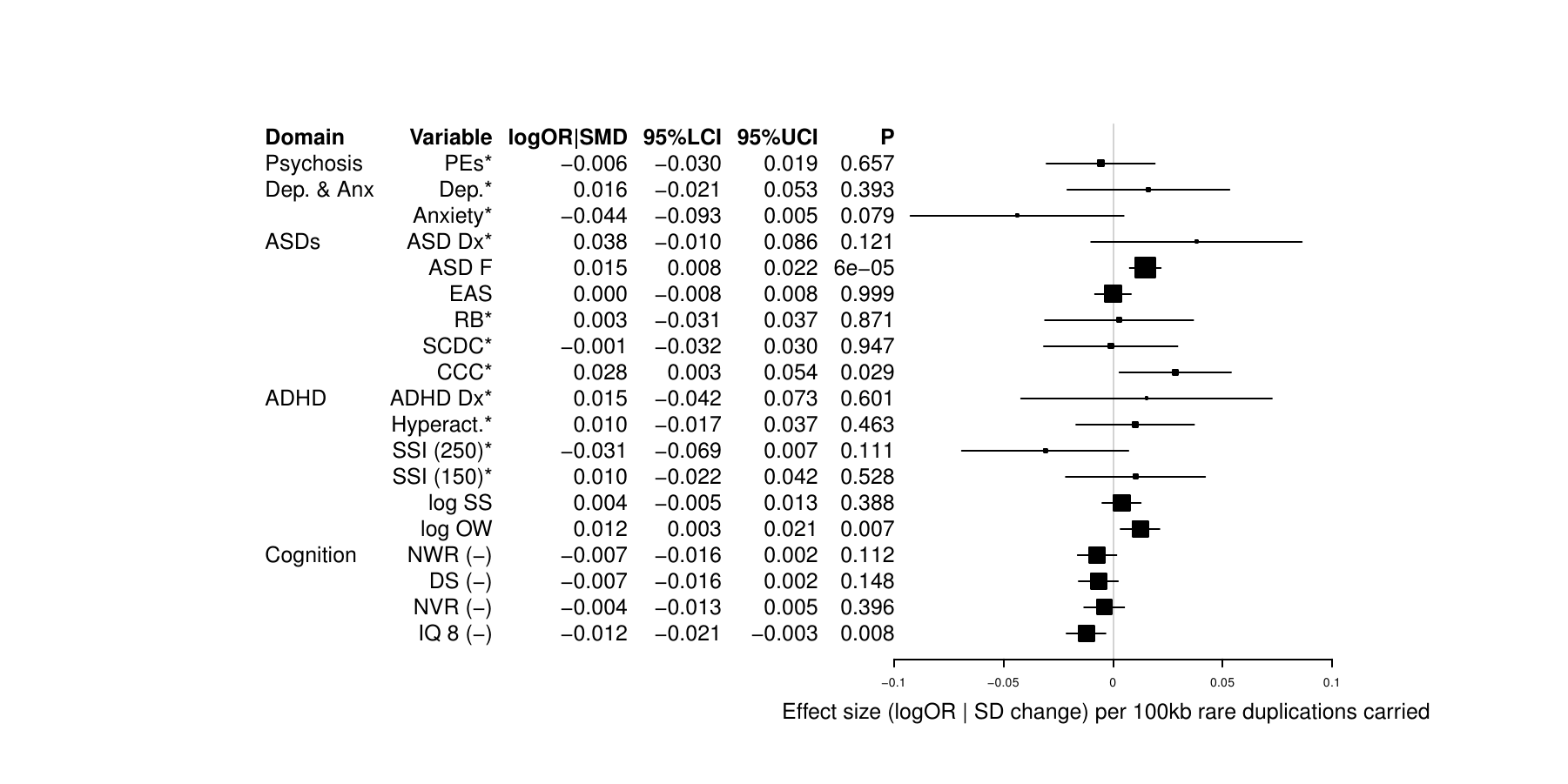


For expansion of trait abbreviations, see Table 1. Other abbreviations: logOR|SMD=effect size (logOR for binary traits [denoted with a *], SD change for continuous traits); LCI/UCI=lower and upper bounds of 95% confidence interval; (-)=lower score is indicative of reduced performance on these metrics (for all other traits, higher scores indicate a reduced performance, or the trait has been dichotomised so that the risk group is coded as ‘1’, control group as ‘0’).

## Supplementary Figure 10

‘Largest CNV carried’ analysis [deletions].


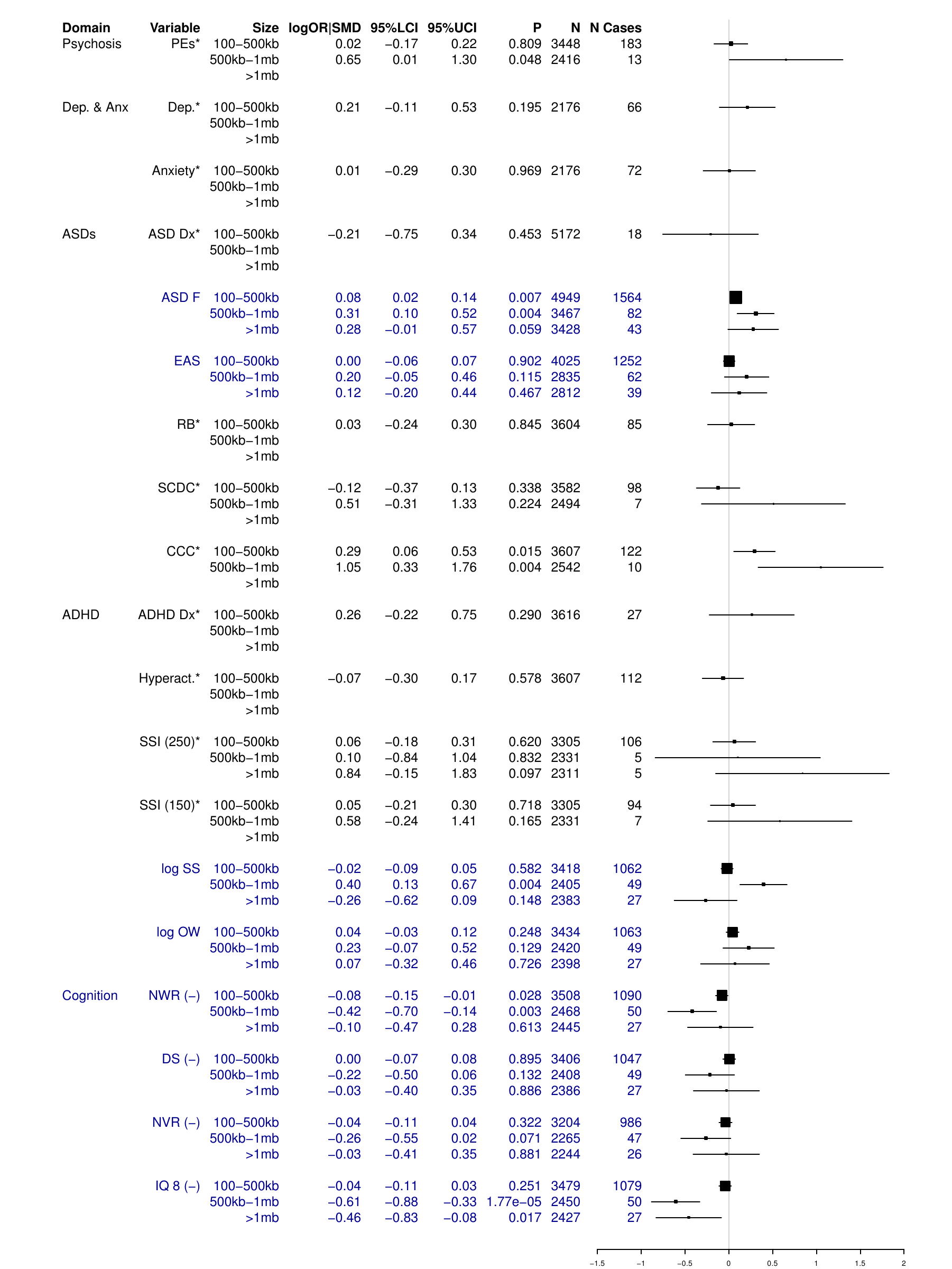


## Supplementary Figure 11

‘Largest CNV carried’ analysis [duplications].


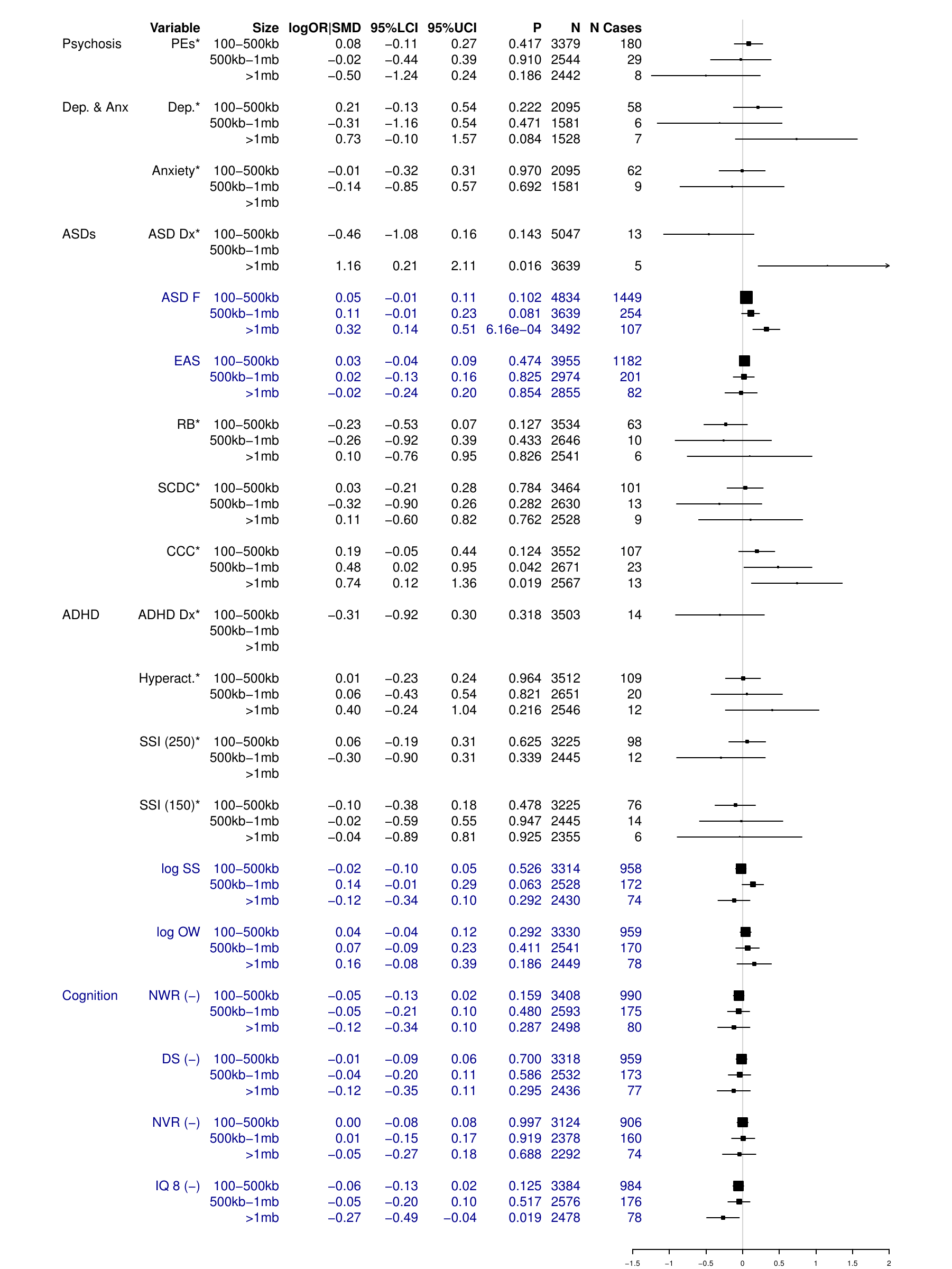


**Supplementary Figures 10 and 11** show the results of the ‘largest CNV carried’ analysis. In this analysis, individuals carrying any of the pathogenic CNVs described in **Table 4** are not included.

Each of the traits in turn is examined in relation to presence (versus no presence) of rare CNVs of increasing sizes. **Supplementary Figure 10** is for deletions, and **Supplementary Figure 11** is for duplications. For expansion of trait abbreviations, see **Table 1**. Other abbreviations: effect size (logOR|SMD) are given as log odds ratios [logOR] for binary traits [denoted with a ‘*’, in black], and as standardised mean differences [SMD, in blue] for continuous traits); LCI/UCI=lower and upper bounds of 95% confidence interval; (-)=for these variables, a lower score is indicative of reduced performance on these metrics (for all other traits, higher scores indicate a reduced performance, or the trait has been dichotomised so that the risk group is coded as ‘1’, control group as ‘0’). Analyses wherein the number of subjects in any cells in the main analysis was below 5 were censored in order to protect confidentiality, concordant with ALSPAC policy.

## Supplementary Figure 12

Sensitivity analysis for the ‘largest CNV carried’ analysis [deletions]. **NB: in this analysis, individuals carrying known pathogenic CNVs are retained.**


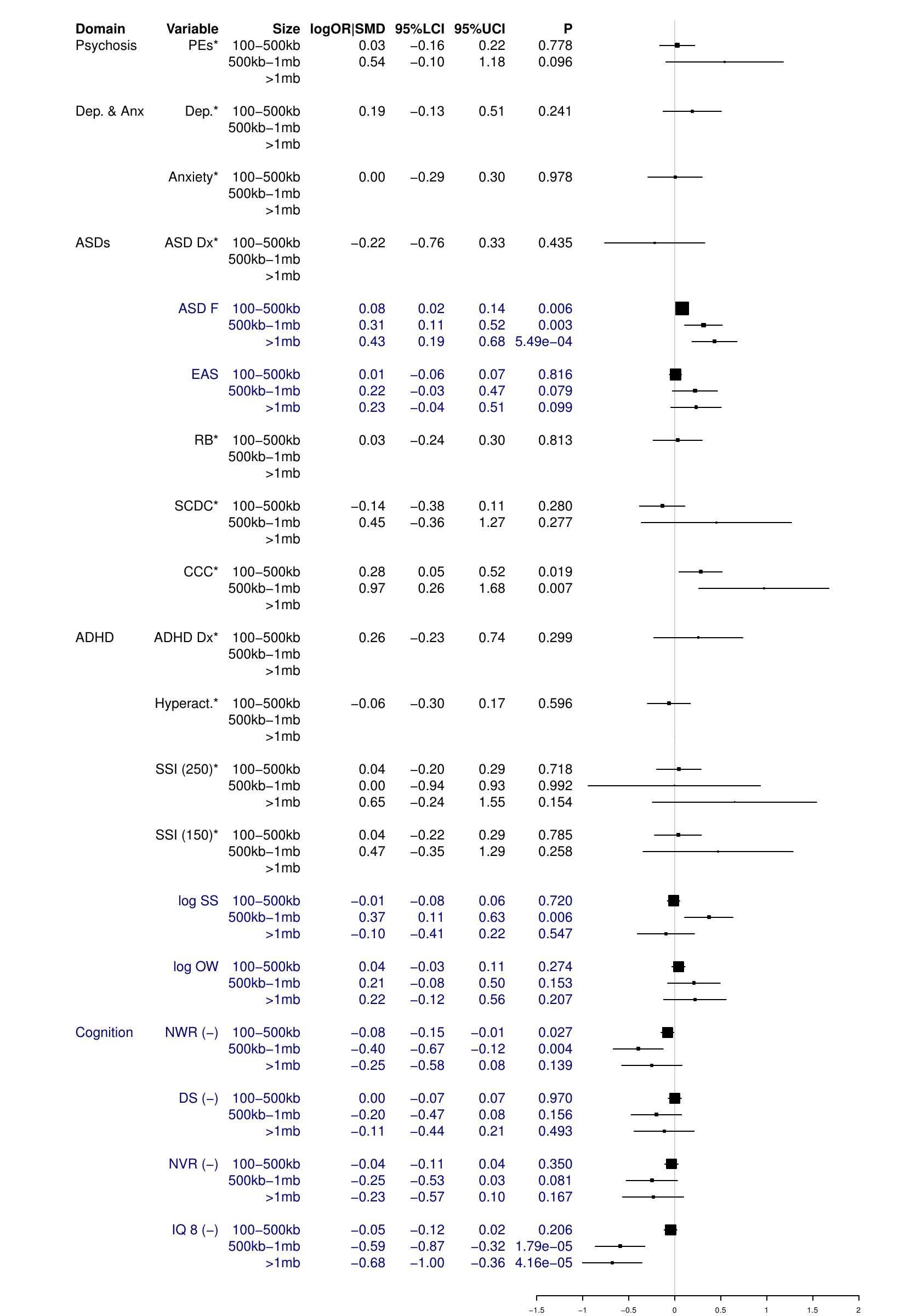


## Supplementary Figure 13

Sensitivity analysis for the ‘largest CNV carried’ analysis [duplications]. **NB: in this analysis, individuals carrying known pathogenic CNVs are retained.**


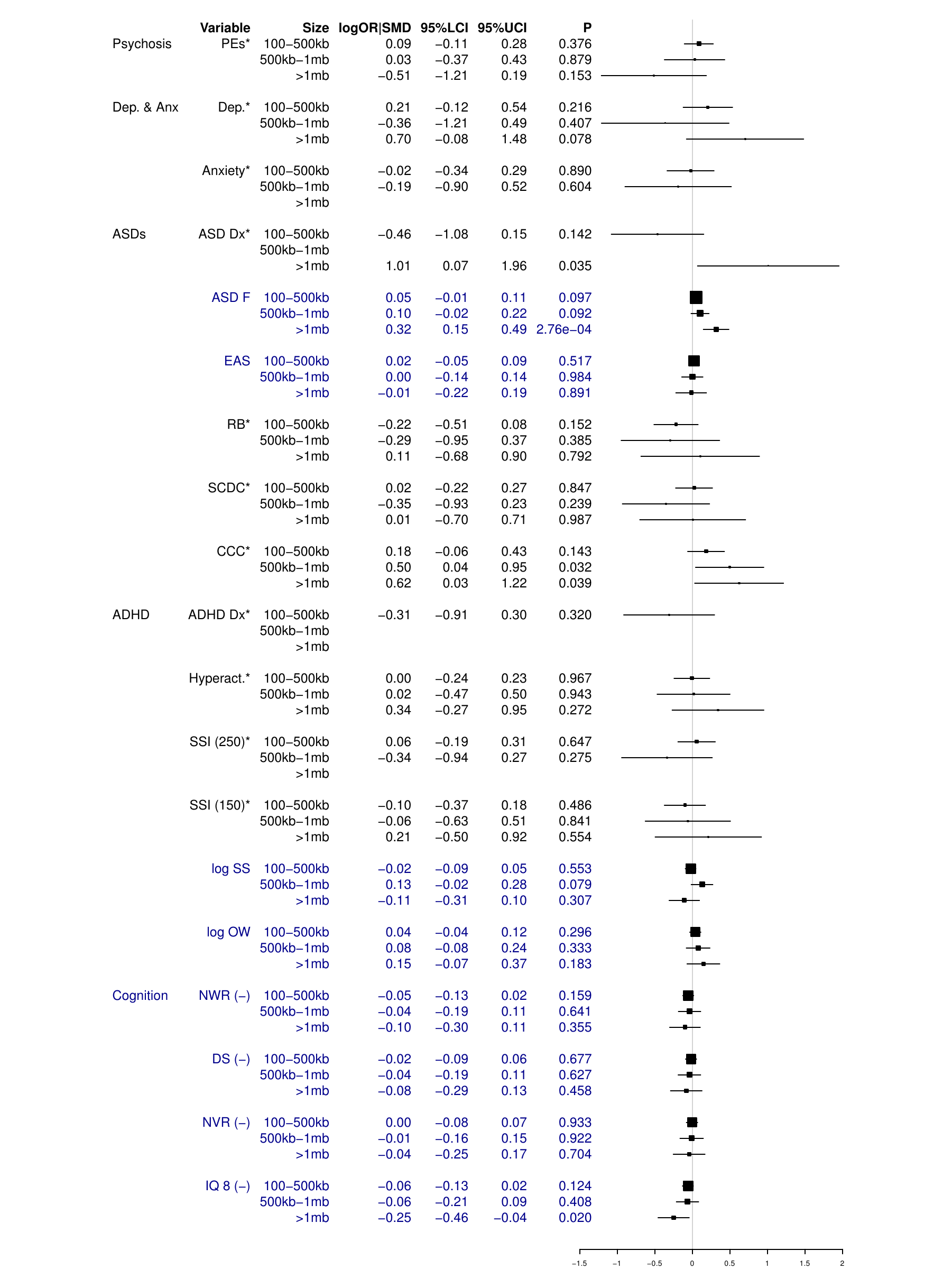


**Supplementary Figures 12 and 13** show the results of sensitivity analysis for the ‘largest CNV carried’ analysis. In this analysis, individuals carrying any of the pathogenic CNVs described in **Table 4** are retained.

Each of the traits in turn is examined in relation to presence (versus no presence) of rare CNVs of increasing sizes. **Supplementary Figure 12** is for deletions, and **Supplementary Figure 13** is for duplications. For expansion of trait abbreviations, see **Table 1**. Other abbreviations: effect size (logOR|SMD) are given as log odds ratios [logOR] for binary traits [denoted with a ‘*’, in black], and as standardised mean differences [SMD, in blue] for continuous traits); LCI/UCI=lower and upper bounds of 95% confidence interval; (-)=for these variables, a lower score is indicative of reduced performance on these metrics (for all other traits, higher scores indicate a reduced performance, or the trait has been dichotomised so that the risk group is coded as ‘1’, control group as ‘0’). Analyses wherein the number of subjects in any cells in the main analysis was below 5 were censored in order to protect confidentiality, concordant with ALSPAC policy.
